# Understanding the Mechanisms of Supported Lipid Membranes Reshaping into Tubular Networks Using Quantitative DIC Microscopy

**DOI:** 10.1021/acs.langmuir.6c00369

**Published:** 2026-05-13

**Authors:** David Regan, Paola Borri, Wolfgang Langbein

**Affiliations:** † School of Biosciences, 2112 Cardiff University, Museum Avenue, Cardiff CF10 3AX, U.K.; ‡ School of Physics and Astronomy, Cardiff University, The Parade, Cardiff CF24 3AA, U.K.

## Abstract

Biological membranes are known to form various structural motifs, from lipid bilayers to tubular filaments and networks facilitating for example adhesion and cell–cell communication. To understand the biophysical processes underpinning lipid–lipid interactions in these systems, synthetic membrane models are crucial. Here, we demonstrate the formation of tubular networks from supported lipid membranes of controlled lipid composition on glass. We quantify tube radii using quantitative differential interference contrast (qDIC) and propose a biophysical mechanism for the formation of these structures, regulated by surface tension and lipid exchange with connected supported membranes. Two lipid types are investigated, namely 1,2-dioleoyl-*sn*-glycero-3-phosphocholine (DOPC) and 1,2-dipentadecanoyl-*sn*-glycero-3-phosphocholine (DC_15_PC), exhibiting a liquid disordered and a solid ordered phase at room temperature, respectively. Tube formation is studied versus temperature, revealing bilamellar layers retracting and folding into tubes upon DC_15_PC transitioning from liquid to solid phase, which is explained by lipid transfer to supported unilamellar layers. A new model system for bilayer tubes is established exposing the biophysics of lipid–lipid interactions governing lipid membrane reshaping into tubular structures, important for our understanding of biological membrane filaments.

## Graphical abstract

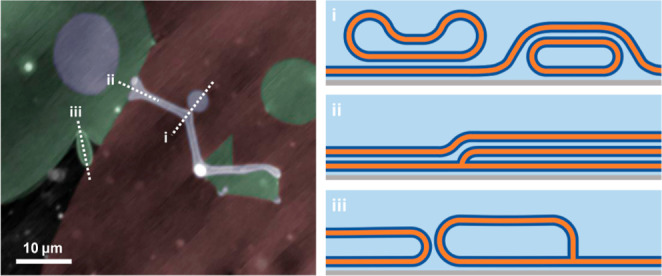

## Introduction

In our endeavor to understand the biophysical properties of cellular membranes, few innovations have had as significant an impact as the development of synthetic membrane model systems.^1^ By allowing the formation of lipid bilayer membranes in controlled geometries with defined compositions, membrane model systems enabled probing aspects of intrinsic membrane behavior in isolation, yielding key insights into cellular activity, including membrane fusion,^2^ membrane fluctuations,^3^ the behavior of membrane proteins,^4^ and the proposed segregation of lipids into phase separated raft domains.^5^

Two of the most commonly used membrane model systems are synthetic unilamellar vesicles^1^ and supported lipid bilayers (SLBs),^1^ which act as analogues for specific types of cellular structures. Small unilamellar vesicles (SUVs), with diameters of tens to hundreds of nanometres, are structurally analogous to biological vesicles, while larger giant unilamellar vesicles (GUVs), which are tens of microns in size, and planar SLBs reconstitute the weak curvature of cell plasma membranes. Alternatively, Langmuir monolayers^6^ and large unilamellar vesicles (LUVs)^2^ can be employed to study membrane biophysics in such planar or gently curved geometries, respectively. However, one type of membrane arrangement found in nature, lipid bilayer tubes, remains difficult to study.

Tubular membranes connect cells, facilitating intercellular transport and communication, such as axons and dendrites of neurons.^7^ Tubular membranes also exist as protruding filaments involved in adhesion and cell movement,^8^ and as cilia to move fluids, propel cells, and form sensory receptors.^9^ Within the cell, tubes are found in organelles such as the Golgi apparatus^10^ and the endoplasmic reticulum (ER).^11^ They form part of a dynamic network including flattened vesicles, called cisternae, in which tubes are remodelled over time, while others are static with nodes where multiple tubes intersect.^12^ New tubes in these organelles are often formed by mechanical interactions of the membrane with the cytoskeleton,^10^ and likewise tubes in model membrane systems tend to be formed from planar membranes in response to external perturbation. Such perturbations may be physical, such as by pulling^13^ or compressing^14^ the membrane, or chemical, such as osmotic gradients over the membrane^15^ or depleting lipids from one leaflet.^16^

Reported model systems for bilayer tubes start from GUVs. For example, a patch of the GUV outer membrane can be adhered to a solid substrate by biotin–avidin binding and then the GUV is pulled away,^13^ leaving a tubular connection between the GUV and the patch bound to the substrate. One can also split a GUV using carbon fibers controlled by micromanipulators, creating two GUVs connected by a tube.^17,18^ While tubular membranes formed in these ways have a controlled length, their free-standing nature limits the range of characterization techniques that can be applied. For example, surface-sensitive methods commonly used to study lipid bilayers, such as atomic force microscopy (AFM), are difficult to implement on unsupported structures. Supported tubular membranes therefore offer a promising alternative.

Straight supported membrane tubes, tens of microns in length, have previously been produced by flow-induced extrusion from dry lipid films on passivated glass surfaces and used to study membrane fission.^19^ In our earlier work,^20^ we showed that spin-coating of a dry lipid film onto a glass substrate followed by gentle humidification can spontaneously generate tubular networks. We assume that the tube formation results from the surface adhesion and complex topology of the bilayer structure created by the spin-coating protocol followed by hydration. These networks remain surface-attached and, once formed, are stable over time scales of minutes to hours, making them well-suited for studying tubular bilayer structures using a wide range of experimental approaches. We also demonstrated that qDIC is a noninvasive optical microscopy technique suited to quantitatively image nanoscale structures, such as lipid bilayer thicknesses and single nanoparticles sizes, with sub-nanometer precision.^20,21^

Building on these earlier studies, here we investigate the formation of supported tubular networks using controlled lipid types, namely DOPC and DC_15_PC, exhibiting a liquid disordered (L_d_) and a solid ordered (S_o_) phase at room temperature, respectively. We apply qDIC to quantify the tube radii over hundreds of tubes, for statistical significance. Beyond sizes, qDIC is sensitive to birefringence, providing information on the three-dimensional spatial arrangement of tubular structures below the optical resolution, and various geometries are revealed. A biophysical mechanism is proposed for the formation of these structures, regulated by surface tension and lipid exchange with connected supported lipid bilayers.

## Materials and Methods

### Chemicals

Lipids without fluorescent labels, namely DOPC and DC_15_PC, were obtained from Avanti Polar Lipids (Alabaster, US) either as powder or predissolved in chloroform, and used without further purification. For fluorescence, 1,2-dioleoyl-*sn*-glycero-3-phosphoethanolamine (DOPE) labeled with ATTO488 was obtained from ATTO-TEC (Siegen, Germany). Chloroform, acetonitrile and 2-propanol were obtained from Sigma-Aldrich (St Louis, US) at HPLC grade or above.

### Spin Coating

SLBs were prepared on glass coverslips using a spin-coating procedure based on that developed by Mennicke and Salditt,^22^ and described in our previous work.^20^

24 × 24 mm^2^ #1.5 coverslips (Menzel-Gläser, Germany) were cleaned by first wiping with acetone-soaked cleanroom paper, and then, to remove any remaining organic material and render the glass surface hydrophilic, etching in a 3:1 (v/v) solution of sulfuric acid to 30% hydrogen peroxide (Piranha solution) at 95 °C for 1 h. Finally the coverslips were rinsed in distilled water, dried under nitrogen flow, and then stored under nitrogen at 4 °C for no more than 2 weeks before use.

Immediately before spin coating, lipid solution was added to the center of the coverslip, which spread out to fully wet the glass surface. The type of solvent was chosen to enhance bilayer formation by spin coating.^23^ For lipids forming a homogeneous L_d_ phase bilayer at room temperature, a 95:5 (v/v) mix of chloroform and acetonitrile was used, while for lipid mixtures that formed an S_o_ phase at room temperature, 2-propanol was used. The solution volumes were chosen to fully wet the surface, and depend on the hydrophilicity of both the solvent and glass substrate, being typically around 300 μL for the chloroform/acetonitrile mixture and 150 μL for 2-propanol.

Spin coating was carried out in air on a Laurell WS-650-23 spin coater, using 30 s at 3000 rpm, with 6 s acceleration and deceleration steps, leaving a dry lipid film on the coverslip. The lipid concentrations used were between 0.8 and 1.2 mg/mL, adjusted to produce lipid films that were mainly unilamellar after hydration, with some gaps and bilamellar regions. The lipids incorporated 0.1 mol % ATTO488-DOPE for fluorescence imaging unless otherwise stated. A detailed discussion of the development of these parameters is given in ref 23.

After spin coating, the lipid-coated coverslips were subjected to a prehydration step, in which they were incubated in a 100% humidity nitrogen environment at 37 °C for 1 h. Prehydration was used to encourage lipid bilayer formation on the surface, without direct contact to bulk liquid water or flow, in order to avoid washing off lipids into liquid volume. Next, lipid-coated coverslips were allowed to cool to room temperature in a 100% humidity nitrogen environment for approximately 1 min. Finally, the lipids were fully hydrated by gently placing the lipid coated face of the coverslip onto a slide with an adhesive gasket filled with room temperature phosphate-buffered saline (PBS) solution of 1× concentration, providing a liquid filled chamber of 120 μm height, in which the reorganization of the lipids completed.

### Imaging

Samples were imaged as previously described.^20^ Briefly, a modified Nikon Ti–U inverted microscope with a 0.75 NA 20× objective (Nikon CFI Plan Apo Lambda MRD00205) and 1.5× tube lens were used, providing a 30× magnification on the camera. Images were acquired using a Hamamatsu Orca 285 CCD camera (sensor size 1344 × 1024, pixel size 6.45 μm, full well capacity 18 ke, read noise 7 electrons, 4.6 electrons per count at zero gain, 12 bit digitization), yielding a corresponding pixel size on the sample of 216 nm. The microscope stage was enclosed in a sealed incubation box connected to a Life Imaging Systems Cube2 heating unit, allowing the temperature at the sample to be controlled; measurements were taken at room temperature unless otherwise stated.

For fluorescence measurements, a metal-halide high pressure lamp (Prior Lumen 200) was used with an epi-fluorescence illuminator (Nikon Ti-FL), filter turret (Nikon Ti-FLC), and a filter cube (Semrock GFP-A-BASIC, excitation 35 nm wide centered at 469 nm, emission 39 nm wide centered at 525 nm). Exposure times were either 1 or 2 s. For differential interference contrast (DIC), a 100 W halogen lamp (Nikon Ti-DH D-LH/LC) was used with a green interference filter (Nikon GIF) to provide an illumination wavelength centered at 550 nm and a full-width at half-maximum (FWHM) of 53 nm. In addition, a Schott BG40 filter was used to remove wavelengths above 650 nm which were not blocked sufficiently by the GIF. The illumination was then transmitted through a de-Sénarmont compensator (a rotatable linear polarizer and quarter-wave plate, Nikon T-P2 DIC Polarizer HT MEN51941) and focused by a 0.72 NA condenser (Nikon CLWD MEL56100) onto the sample. We used Nikon N2 prisms with a shear separation of *s* = 238 ± 10 nm (a MBH76220 slider for the objective and a MEH52400 module in the condenser). For qDIC analysis, pairs of DIC images were taken, with the angle of the linear polarizer set to ± θ, with θ being either 13° or 15°. Each DIC image was averaged over 100 acquisitions of 100 ms exposure time.

### Quantitative DIC

Quantitative differential interference contrast (qDIC) allows the generation of quantitative phase maps from pairs of standard DIC images, as detailed in our previous works.^20,21,24^ Briefly, a pair of DIC images is taken at opposite de-Sénarmont polarizer angles (*I*_+_ at + θ and *I*_–_ at – θ), which are then used to produce a contrast image

1 $$I_{c}=\frac{I_{+}-I_{-}}{I_{+}+I_{-}}$$

from which the phase difference in the sample δ can be retrieved using

2 $$I_{c}=\frac{\sin\left(2\theta \right)\sin\left(\delta \right)}{\cos\left(2\theta \right)\cos\left(\delta \right)-1}$$

which can then be rearranged^21^ into an analytical expression to determine δ from *I*_C_ and θ. The quantitative phase map ϕ­(**r**) of the sample is then found by integrating δ­(**r**) along the shear direction with a Wiener filter using a signal-to-noise parameter κ which is creating a high-pass cutoff in spatial frequency.^21^ To reduce the striping resulting from this integration, a minimization algorithm penalizing spatial gradients can be used, as shown in Regan et al.^20^ and Section S1.

## Results and Discussion

### Bilayer Structures of DOPC Lipids

As introduced in our earlier work,^20^ spin-coated lipid films form thin “branch-like” lipid networks on the glass surface upon hydration, in addition to planar lipid bilayers. These networks form spontaneously from spin-coated lipid films. An example of a supported DOPC lipid bilayer stack is given in Figure 1, showing the phase difference δ­(**r**) in (a), the reconstructed phase ϕ­(**r**) in (c), and the fluorescence in (b). A detailed discussion of qDIC images characterizing the planar bilayers was given in our earlier work.^20^ Here, we focus on the characterization of the tubular structures. As seen in Figure 1, we observe that these structures are connected to unilamellar planar bilayer regions (see midrange gray contrast), and exhibit a bright contrast, similar to that of a bilamellar stack.

**Figure 1:**
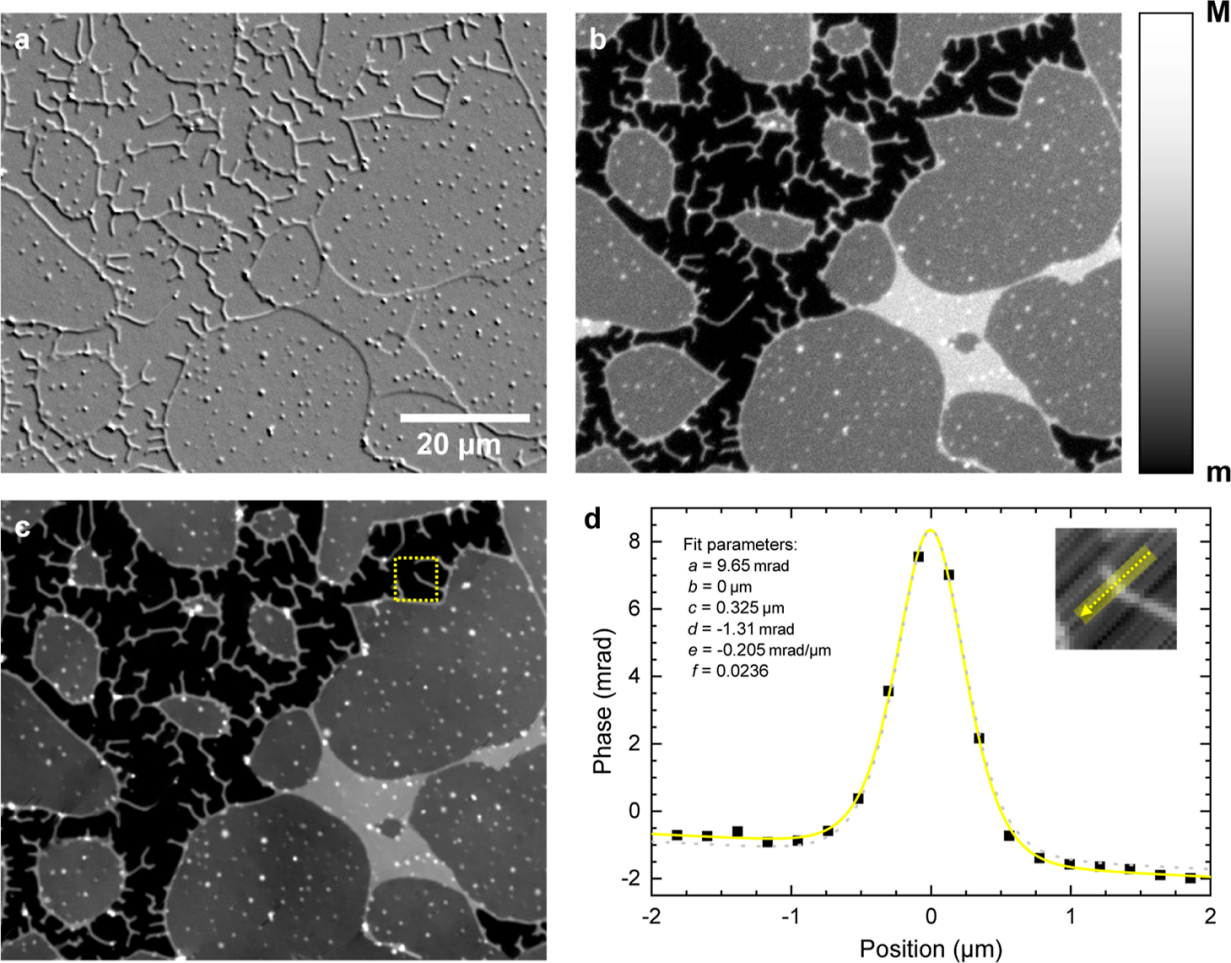
(a) A qDIC δ­(**r**) image showing a region of a spin-coated DOPC SLB stack in which tubular networks have formed, scaled from *m* = −6 mrad to M = +6 mrad. (b) The corresponding fluorescence image, scaled from *m* = 80 to *M* = 260 counts. (c) The same region shown as a minimized qDIC phase ϕ­(**r**) image, scaled from *m* = 0 mrad to *M* = 20 mrad. (d) The phase profile across a tube; the line cut is shown in the inset. The yellow line is a fit using eq 3 with the parameters shown, and the gray dashed line shows the fit function using *f* = 0, i.e. without birefringence.

To extract quantitative structural information, we take line profiles perpendicular to the tube’s direction,^20^ as illustrated in Figure 1d. To reduce the noise in these cross-section profiles, we average over a width of four to eight pixels (0.9 to 1.7 μm). In order to minimize the influence of qDIC integration artifacts, we only take cross sections that are nearly parallel to the DIC shear direction (further discussion on integration artifacts is provided in our earlier works^20,21^ ). These cross sections are then fitted using

3 $$t\left(x\right)=a\left[{\mathrm{sech}}^{2}\left(\frac{x-b}{c}\right)+f\;\tanh\left(\frac{x-b}{c}\right)\right]+d+ex$$

where the sech^2^ term represents a structure which is smaller than the optical resolution of our imaging system, thus appearing as a peak in the phase profile. The parameter *a* is the peak amplitude, *b* is the peak position, and *c* the peak width giving a measure of the tube diameter convolved with the optical resolution. Local background gradients in the image are taken into account by the linear term *d* + *ex*. The tanh term with relative amplitude parameter *f* models the effect of birefringence, as detailed later.

An example of a phase cross-section through a tubular structure and the corresponding fit are shown in Figure 1d. By integrating the area contained within the curve, we can quantify the amount of bilayer material in the cross-section. The integral of the sech^2^ term is 2*ac*. We can convert this value into a corresponding bilayer length, dividing by the optical thickness of a single bilayer, which is 5.73 mrad as determined in our previous work.^20^ For the structure shown in Figure 1d, we find a lipid bilayer length of (1096 ± 57) nm in the cross-section, with the uncertainty giving the 95% confidence interval of the fit. Attributing the observed structure to a circular tube geometry yields a radius of (174 ± 9) nm, consistent with its resolution limited appearance. An alternative configuration consisting of a stack of two flat bilayers would require a width exceeding 500 nm to contain this amount of lipid, while the peak width in the fit in Figure 1d is close to resolution limited (*c* = 325 nm).

Further evidence for the tubular geometry of the membrane is provided by the slight mismatch of the background level on either side of the peak in the phase profile. This is modeled by the tanh term in eq 3, with the prefactor *f* describing the relative amplitude of the step. The scale of this effect is illustrated in Figure 1d by the gray dashed line showing the fit with *f* set to zero. Such changes in phase around the peaks can be explained by the birefringence of the lipid bilayers, which have an ordinary refractive index (*n*_0_ = 1.445) for light polarized in the bilayer plane, and an extraordinary index (*n*_e_ = 1.460) for light polarized perpendicular to that plane.^25^ In DIC, the two sheared beams are polarized along and orthogonal to the shear. In a tubular geometry, the bilayer is oriented partly out of plane, so that for tubes aligned orthogonal to the shear, one of the beams is polarized along the tube, and thus experiences only *n*_0_, while the other is polarized orthogonal to the tube, and thus experiences a mixture between *n*_0_ and *n*_e_, depending on tubular geometry. This results in a phase difference between the two beams, which, after integration, provides a phase step. Notably, this effect does not occur for planar bilayer geometries, where both DIC polarizations are in-plane. Hence, the observed phase-step provides evidence for the tubular geometry.

Extending the region shown in Figure 1, we observe tubular structures of different contrast, as shown in Figure 2, indicating a variation in tube size. We fitted tube cross sections along the colored lines, and similarly over more fields of view (see Section S9). Figure 3e shows the resulting phase steps as a function of tube circumference and Figure 3f the peak amplitude as a function of width. Assuming that the tubes are unilamellar with circular cross-sections, the mean and standard deviation of the radii are 181.8 ± 47 nm, over *n* = 125 measurements. The mean diameter of 364 nm is close to the diffraction limit, consistent with the fitted width. The radii of these supported tubes are similar to those of tubes pulled from GUVs,^17^ but are larger than the typical radii of tubules in cellular organelles, which tend to be below 100 nm.^10^ To understand these differences, the following considerations can be made.

**Figure 2:**
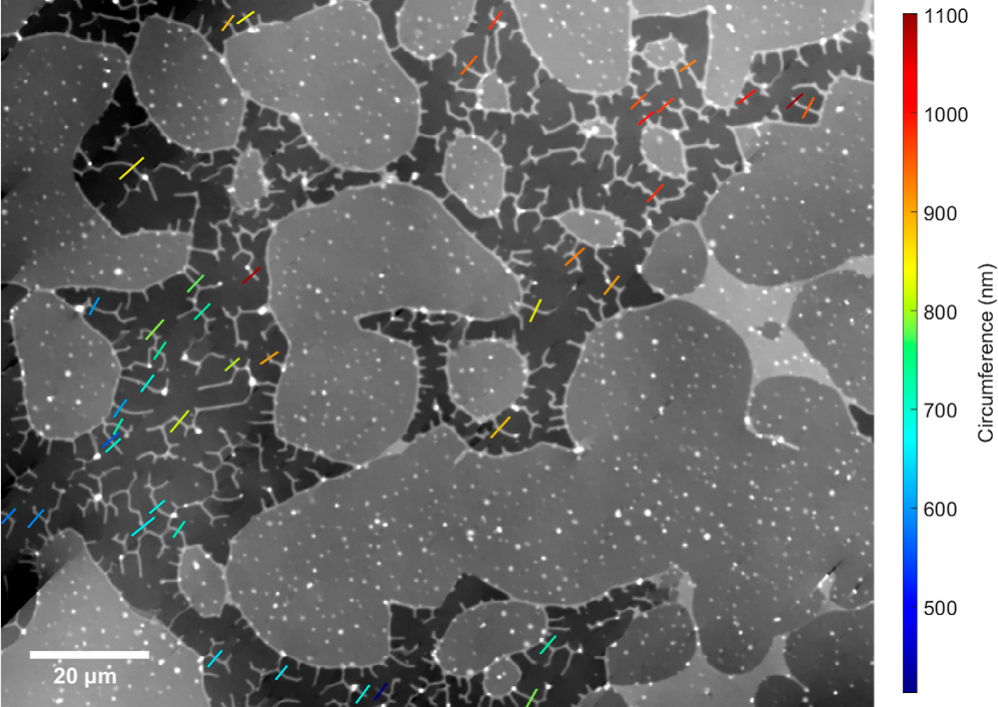
Minimized qDIC phase image containing the region shown in Figure 1, scaled from −18 to 6 mrad. Lines show measured profiles across tubes, with a color encoding the resulting tube circumference on a scale as indicated.

**Figure 3:**
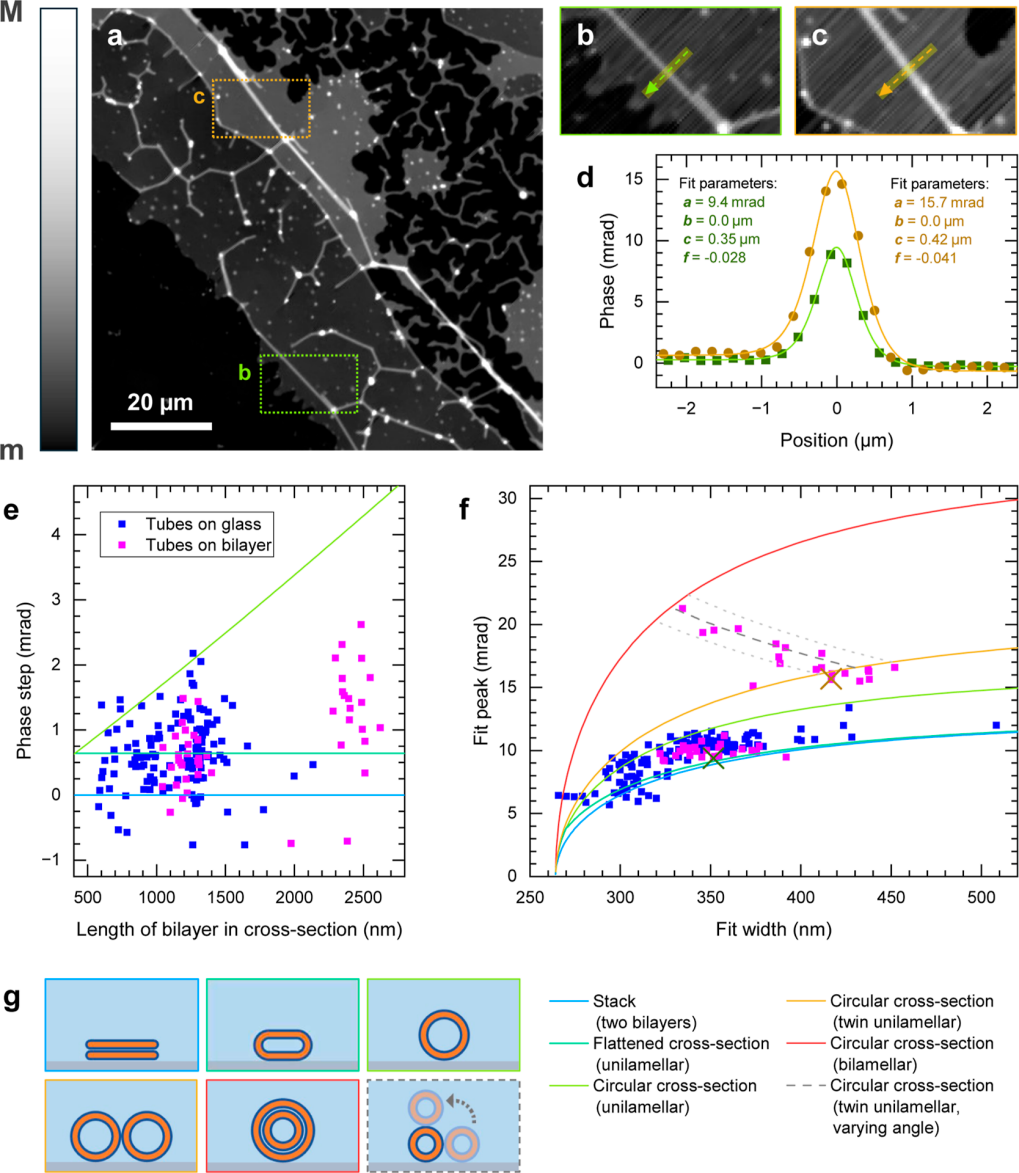
(a) Minimized qDIC phase image showing a region with tubes formed on top of other bilayers, as well as tubes formed on glass, scaled from *m* = 0 mrad to *M* = 32 mrad. (b,c) Line cuts through two different tubes, with phase profiles and fits shown in (d). (e) Phase step 2*fa* due to bilayer birefringence versus the length of the bilayer cross section for tubes formed on glass (blue) and on other bilayers (magenta). Colored lines show the expected phase step for different cross-sectional shapes (see upper row of g) as a function of circumference. (f) Fitted peak amplitude *a* against width *c*, from the fitting to the DOPC tube phase profiles, with simulated curves for different cross-section shapes shown as colored lines. The positions of the points corresponding to the phase profiles shown in d are denoted by crosses. (g) Illustration of the different cross-section shapes simulated in (e,f), with corresponding frame color.

For a tube with a circular cross-section, the radius *r* is determined by the balance between the bending rigidity of the bilayer, η, and the tension in the tube, σ, resulting in^10^

4 $$r=\sqrt{\frac{\eta }{2\sigma }}$$

The bending rigidity depends on the lipid species present, and on the relative distribution of these lipids between the two leaflets of the bilayer. Our system is almost exclusively formed from phosphocholine (PC) lipids which have a low spontaneous curvature.^26^ In contrast, biological membranes often contain curvature inducing proteins,^10^ as well as significant proportions of lipids with high intrinsic curvature such as phosphoethanolamines (PE),^27,28^ which provide finite equilibrium radii *r*_0_, yielding

5 $$r^{-1}=\sqrt{\frac{2\sigma }{\eta }}+r_{0}^{-1}$$

Indeed, it has been previously shown for tubes pulled from GUVs that when tubes can draw from reservoirs of higher spontaneous curvature PE lipids, tube diameters are reduced.^26^

The surface tension in the tubes is governed by the overall sample geometry and the membrane–substrate interactions. Comparable model systems to ours consisting of linear supported membrane tubes produced by flow-induced extrusion have radii in the 10 to 40 nm range,^19^ considerably smaller than what we observe with similar lipid compositions, suggesting that our system is under lower tension. With qDIC we can explore tension variations within our system, since for a constant lipid composition (and therefore constant η), variations in tube radius must correspond to changes in tension. In Figure 3e, we clearly see different groupings of circumference values in the data, with clustering around 650, 950, and 1300 nm, which correspond to measurements taken in different sample regions. Even within single fields of view, such as shown in Figure 2, we can see clustering of different circumference values; in the bottom left corner we see tubes with lower circumference values corresponding to the 650 nm cluster, while the tubes in the upper right belong to the 950 nm cluster. This indicates that the tube circumference reports variations in membrane tension over ranges of a few hundred microns within the SLB (see also Supporting Information, Figure S3).

In addition to the short, branched tubes protruding from the edges of the bilayer stack, as seen in Figure 1, we observe tubes with long straight sections. An example is shown in Figure 3a where a structurally distinct tubular network has formed attached to a SLB band extending from top left to bottom right. These tubes appear to be above rather than below the bilayer, as the latter would result in the bilayer bending around them, which could be seen as a increased optical thickness. They have straight segments often over 10 μm in length, compared to a few μm lengths observed for tubes on glass, indicating that the tube-substrate interaction has a major role in shaping the tube networks. We attribute the shorter tube lengths on glass to adhesion points on the glass surface which create corners or end-points in the network. These are screened in bilayer-coated regions, allowing for long straight tubes to be created by the surface tension.

The results of fits to the tubes on top of other bilayers are shown in Figure 3e as magenta points, again showing clustering of circumference values. The lower cluster has a mean circumference of 1213 nm and a standard deviation of 90 nm (*n* = 27), which is similar to the circumference of the tubes on glass in the same region (1332 nm, standard deviation 161 nm, *n* = 66). This suggests that both types of tubes are under similar tension, as expected if they are formed from a contiguous bilayer.

The population of higher circumference tubes centered around 2400 nm would have a diameter of about 800 nm, assuming a unilamellar tube with a circular cross-section, which should be resolvable in our qDIC microscope. An example of one of these tubes is given in Figure 3c, with the corresponding phase profile and fit shown in orange in Figure 3d. For comparison, a lower circumference tube is shown in Figure 3b, and in green in Figure 3d. While the fitted width for the tube in Figure 3c of 417 nm is larger than the close to resolution limited width of 352 nm for the tube in Figure 3b, it is much less than expected for a 800 nm diameter.

We therefore considered alternative tube cross-sections illustrated in Figure 3g. We note that the fit parameters of the qDIC phase profiles, specifically the peak amplitude *a*, width *c*, and the phase step 2*fa*, are dependent on the tube geometry. To identify different tube geometries, we calculated the expected phase step due to the birefringence for different cross-sections, shown as colored lines in Figure 3e. We also simulated spatial phase profiles neglecting birefringence, and applied eq 3, see Section S2 for details. The resulting peak amplitudes and widths are shown in Figure 3f, for a nontubular arrangement of two stacked bilayers (blue line), a flattened unilamellar tube having an edge radius of 64.8 nm (dark green line), a unilamellar tube (light green line), a pair of unilamellar tubes running side-by-side (orange line), and a bilamellar tube with circular cross-section (red line). We can see that the peak amplitude for tubes formed on glass generally is located between the simulation of a unilamellar flattened bilayer cross-section and that of a circular cross-section, corresponding to a slightly squashed configuration of the membrane, which could be caused by substrate adhesion. We note that a slight defocus of the images can lead to an increased width, which might explain the data points below the bilayer stack simulations. Flattened membranes show little differences to two stacked bilayers when the cross-section is dominated by the flat part. Yet, these two configurations correspond to distinct predictions regarding the phase step due to the birefringence (Figure 3e), indicating that with qDIC we could distinguish these cases. The birefringence data generally resides between that of a circular cross-section (green line) and a flattened geometry (dark green line). However, the error in the phase step (dervived from the 95% confidence intervals of the fit parameters *a* and *f*) is around ±0.7 mrad, providing a significant spread of the data.

Coming back to the peak amplitude in Figure 3f we see that the tubes formed on top of other bilayers (magenta points, see Figure S25 for the corresponding image with measurement positions) are split into two populations. One population is consistent with the tubes formed on glass, suggesting that they share a common structure. The other population has significantly higher peaks than expected for a unilamellar bilayer with circular cross-section (light green line), but lower than a bilamellar circular cross-section (red line). Notably, the population is stretched along a band between the red line and the orange line representing two side-by-side circular tubes of equal radii, illustrated in Figure 3g. The band is well described by a pair of attached circular cross-section tubes with varying arrangement from side-by-side to stacked, having radii given by the mean of the isolated tubes (193 nm, dark gray dashed line) or one standard deviation above or below, 207 or 179 nm (lighter gray dashed lines). Thus, qDIC indicates that this population of tubes is not bilamellar, but consists of pairs of tubes wrapped around each other, like a twisted pair cable. This interpretation is consistent with the variation along the tubular structure shown in Figure S26 as example.

An independent verification of the geometry with other techniques is challenging, as the structures are in liquid, soft, and not stable over longer time. A correlative measurement using AFM could be problematic, as the AFM tip perturbs the DIC measurement, and furthermore the pressure applied on the tubes by the tip could result in deformation, while internal structures would not be visible. Cryo-electron microscopy would require rapid high-pressure freezing, which is likely to distort the structure by mechanical shock and lipid phase transition. Instead, the qDIC method is specifically suited to study lipid bilayer structures, owing to its noncontact geometry compatible with intact samples. Notably, we have demonstrated the quantitative accuracy of our qDIC method by comparison with AFM and TEM on calibration polystyrene beads in our previous work in Hamilton et al.^21^

Beyond characterizing the tubes themselves, it is interesting to investigate the lipid structure at the point where tubes meet the planar supported bilayer regions of the sample. We see in Figure 1 that the unilamellar bilayer regions from which the tubular networks protrude appear to be surrounded by thicker borders running along their perimeter, visible in fluorescence (Figure 1b) and qDIC phase (Figure 1c) as lines of higher contrast. An example of a phase profile over the border of a unilamellar region is shown in Figure 4, showing a step due to the transition from bare glass to a single planar bilayer, and a peak at the edge.

**Figure 4:**
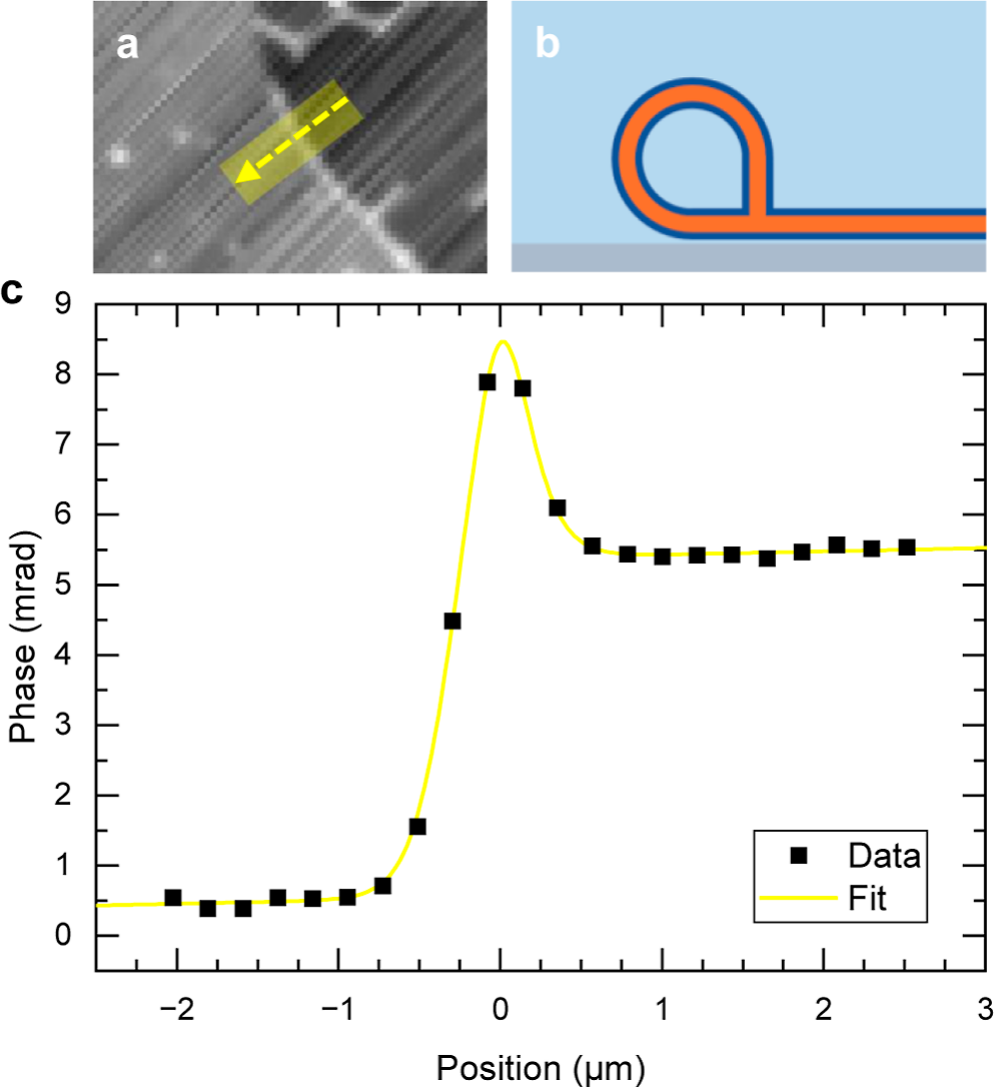
(a) Phase image of a unilamellar region with a border of higher optical thickness. (b) Illustration of the proposed cross-section topology at the border, with a bilayer triple junction structure. (c) Phase profile along the region indicated in a, and fit.

We have fitted the phase profile over this border using eq 3, modified by replacing the position *b* in the tanh term with an additional parameter *g*, to allow for independent positions of the bilayer edge and the center of the tubular structure described by the sech^2^ term, noting that the tanh term is dominated by the optical thickness of the planar lipid bilayer rather than the tubular birefringence. We find in these fits that the mean difference between the position of the border and the bilayer edge *b*–*g* is zero within error (32 nm with a standard error of 49 nm), indicating that they are physically linked in a specific way. The mean bilayer length contained within the tubular border structure is found to be (666 ± 55) nm (*n* = 24, error given is the standard error). We propose that the edge tubular structure contains a bilayer triple junction, as illustrated in Figure 4b. We note that the length of the tubular border structure is (77 ± 7)% of the observed mean bilayer length (861 ± 21) nm (*n* = 49) of the tubes connected to the borders, commensurable with the hypothesized geometry which is missing up to one quadrant of a circular shape. It would be interesting to use simulations to predict the border structure including the relative length, to compare with the experimental results.

### Bilayer Structures of DC_15_PC Lipids Across the Phase Transition

DC_15_PC bilayers are in the S_o_ phase at room temperature and have a phase transition between S_o_ and L_d_ at a temperature^29^ of *T*_*m*_ = 33.7 °C. The S_o_ phase has a higher thickness and a higher areal density of lipid molecules than the L_d_ phase. SLBs using DC_15_PC were prepared as explained in Materials and Methods, by prehydration at 37°, followed by cool-down over a few minutes and final hydration at room temperature. These samples were then mounted in the Ti–U microscope which was preheated to 37 °C. After mounting, samples were allowed to stabilize for at least 10 min, and then the temperature was slowly lowered, typically in 0.3 °C steps with 10 min intervals. An example of the resulting shape transformation occurring in the membrane bilayers, including the formation of tubular structures, is shown in Figure 5a–f.

**Figure 5:**
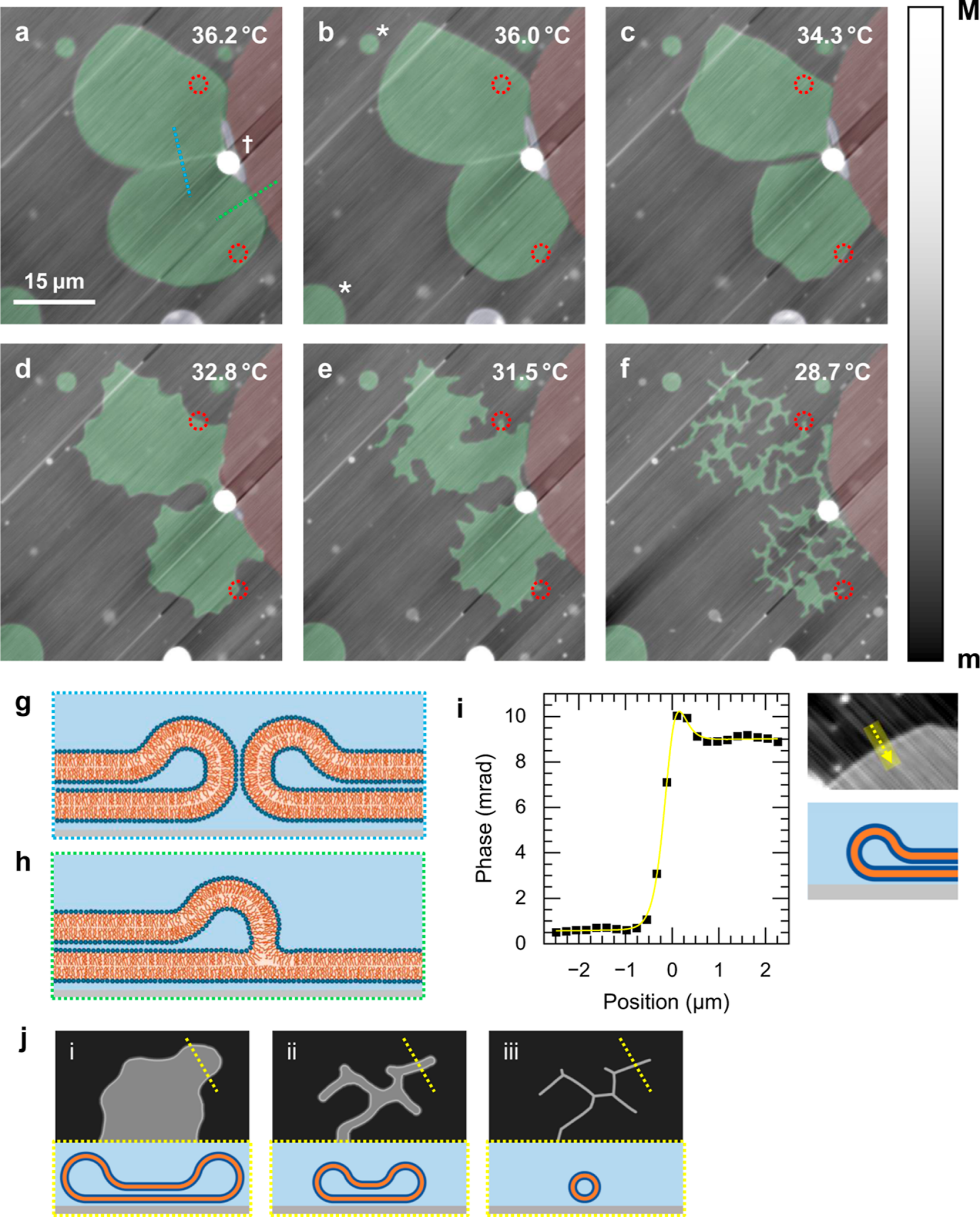
(a–f) qDIC phase images showing shape changes in a DC_15_PC bilayer during cooling (*m* = −33 mrad, *M* = 23 mrad). False color indicates lamellarity: unilamellar (red), bilamellar (green), and multilamellar (blue). Red circles mark selected membrane–surface pinning points. The images without markup are given in Figure S19. (g,h) Illustrations of bilayer arrangements along the blue and green dashed lines in (a). (i) Phase profile across a bilamellar edge and its fit, as in Figure 4, with corresponding phase image and structural illustration. (j) Illustration of the mechanism of bilamellar-to-tubular rearrangement, shown as plan-view and cross-section.

In Figure 5a, a section of the bilayer stack is observed after it has been heated in the microscope to 36.2°, as measured at the sample. Using the qDIC phase information, we identify a large bilamellar region (highlighted in green) attached to a unilamellar region on the right (highlighted in red). The bilamellar region is comprised of two separate parts, as indicated by the line of higher phase contrast at the intersection between the two. The existence of this boundary suggests that at the edges of these bilamellar parts, the bilayers are folding back onto themselves, as illustrated in Figure 5g. This folding prevents the two patches from merging, and can be quantified in the phase profile over the bilamellar patch edge. Figure 5i shows a line cut from elsewhere in the field of view connected to the same unilamellar patch (see Figure S21) showing a clear peak in the phase, where the bilayer is oriented vertically, which then levels off, as expected for a stack of two bilayers. The bilamellar regions can therefore be considered as deflated vesicles sat on the surface, analogous to the cisternae in the ER. By integrating the fit in Figure 5i, we find that the peak contains 558 nm total lipid length; assuming a semicircular cross-section, this would correspond to a radius of curvature of 178 nm.

As the sample is cooled (Figure 5b–f), both bilamellar regions gradually contract. Initially, the edges recede uniformly as the area of each bilamellar patch shrinks, but eventually the edges become pinned at certain points, giving the patches first a polygonal, and then a jagged appearance. Two examples of such pinning points are indicated as red circles on Figure 5a–f. As the area of the bilamellar regions contracts further, more of these pinning points are encountered by the receding edge, until finally the bilamellar regions have completely reorganized into networks of flattened tubes linking these pinning points together. This process is illustrated in Figure 5j, which shows the transition from a bilamellar patch (i) to tubes (iii). A further example of this tube forming process is shown in Figure 6. More details are given in Section S5.

**Figure 6:**
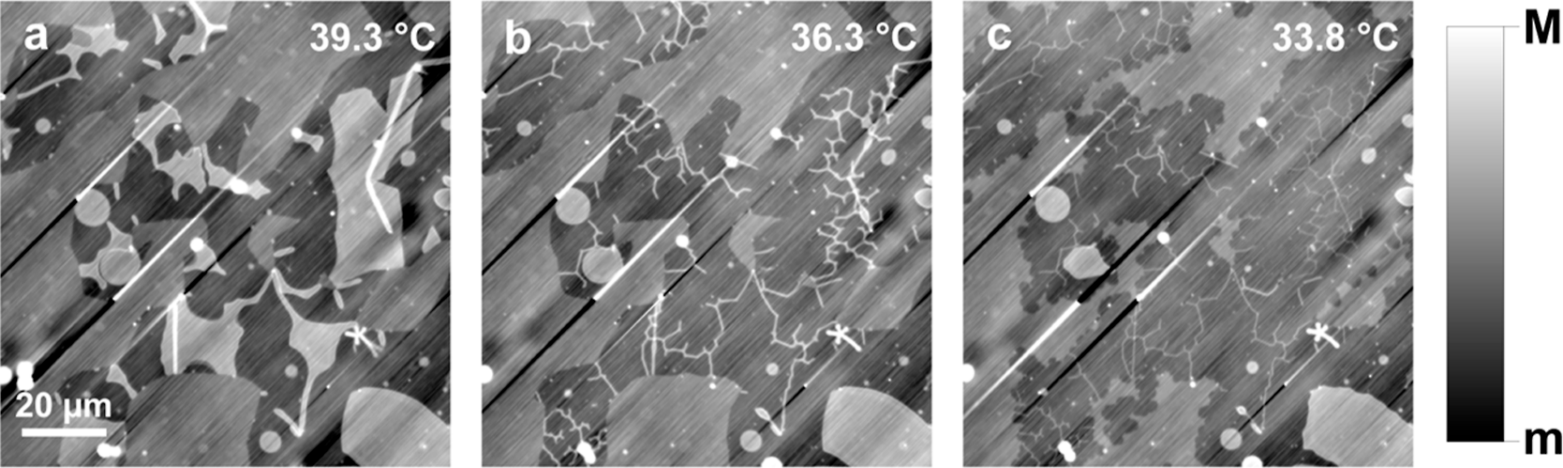
(a–c) qDIC phase images showing tube formation in a DC_15_PC bilayer sample during cooling (*m* = −3 mrad, *M* = 20 mrad). Note the different tube radii visible in (c), suggesting different levels of tension in different tubular networks.

We also note that while the bilamellar regions shrink, the position of the boundary between the unilamellar and bilamellar regions remain mostly unchanged. In other words, the bilamellar regions do not themselves become unilamellar, but rather the two bilayers in the bilamellar regions contract together from the outer edge inward. We therefore suggest that the structure at the unilamellar-bilamellar boundaries (see the green dashed line in Figure 5a) is a triple junction structure, as illustrated in Figure 5h, reminiscent of those we showed previously in Figure 4. However, the observation that its position seems fixed is interesting, as one could expect that such a triple junction could move to reduce the double bilayer area. Relevant considerations are the enclosed water volume in the double bilayer, and the local geometry. Simulations of this situation would be helpful but seem not to be available.^30,31^

The observed tube formation mechanism seems analogous to the earlier mentioned methods which produce tubes by attaching part of a GUV surface to a solid substrate, and then physically pulling the GUV away, leaving a membrane tube connecting the GUV to the attachment point.^13^ Interestingly, in our system no additional molecules have been introduced to bind the membrane lipids to the glass substrate at particular sites, nor have we applied surface passivation to prevent the tubes collapsing into supported bilayers^19^ or otherwise limited the tube-surface interaction. The effect observed is therefore driven purely by local variations in the interaction between the membrane and the hydrophilic glass surface.

Given that the tube formation shown in Figure 5 occurs during the depletion of lipid area from the bilamellar regions of the membrane, in the following we discuss what drives this depletion. First, we note that bilamellar regions which are not connected to unilamellar regions generally do not undergo significant shape transformations during cooling. This is exemplified by the isolated bilamellar patches indicated by the asterisks in Figure 5b, which maintain a constant area. The amount of material in these patches can be quantified (see Section S6). After subtracting a quadratic fit to the phase profile, accounting for background around each patch, and then integrating, we find their phase area. For the top-left and bottom-left patches, we find phase areas of (90.9 ± 6.0) mrad μm^2^ and (893.0 ± 10.3) mrad μm^2^ at 36.2 °C, and (101.2 ± 5.8)­mrad μm^2^ and (974.1 ± 7.8) mrad μm^2^ at 29.2 °C, respectively.

Together, these observations indicate that bilamellar regions need to be connected to unilamellar regions for tube formation to occur, and suggest lipid migration from bilamellar to the unilamellar regions upon cooling. As a driving mechanism for this migration, we showed in our previous work that strong bilayer–surface interactions lead to stretching of the upper leaflet of the first bilayer.^20^ The surface-attached bilayers showed an about 10% reduced thickness, corresponding to an areal stretching of the upper leaflet by 20%, assuming that the reduction is due only to changes in the upper leaflet. This expansion corresponds to a tension of around 25 mN/m, using a monolayer area elasticity of 120 mN/m for DOPC,^32^ which provides an estimate of the adhesion strength of the lower leaflet to the glass. We note that 20% stretch is well above a typical free bilayer rupture threshold and would correspond to a nominal tube radius of 1.2 nm using eq 4. We can thus conclude that the surface adhesion is much stronger than the surface tension in the observed tubes. Consequently, we can expect that the surface area of the lower leaflet of unilamellar regions is fixed, so that upon cooling more lipid is required to cover the upper leaflet of the surface attached bilayer considering the higher areal density of the S_o_ phase. This results in lipid migration from the upper leaflet of the upper lamella of the bilamellar regions flowing to the upper leaflet of the unilamellar region via the triple junction. Exchange of lipids between the leaflets of the bilamellar region is expected to equilibrate the resulting unbalance over time. In turn, bilamellar regions get gradually pulled off the surface from the edges, unless they are strongly anchored, for example in small cavities of the etched glass. These positions form the end or corner points of the remaining tubular structures.

Interestingly, the rather constant areas of the unilamellar patches suggests that while lipid transfer into these regions occurs to compensate the increasing surface density of lipids during cooling in the upper leaflet, the surface attached area of the lower leaflet stays constant. This indicates that the surface interactions established during spin coating are significantly stronger than interactions after hydration, which is plausible due to the screening of electrostatic interactions by water. The area of the unilamellar regions are thus determined by the initial spin-coated configuration of the lipid film, and upon formation of bilamellar structures with rounded edges, the lower leaflet can be stripped off the surface, as seen during the tube formation. The development of a corresponding stronger stripping force resembles the action of a mechanical pulley, doubling the tension for bilamellar edges.

The presence of a direct contact of the lower lipid leaflet to the glass is a consequence of preparing the bilayers by spin coating, where lipids come in direct contact with the polar glass surface in the absence of water. For bilayers created by SUVs rupturing onto a polar glass surface, this is not the case, as the polar headgroups are already coordinated with water when arriving at the surface, which leads to the widely reported water interlayer of some 1 nm thickness.^33^ To support the hypothesis of direct contact, we show in the supplement Section S4 an experiment where we found that over time, this contact can be lost gradually in a medium with 2 mM Tris. We observe a detachment front, as an increase of fluorescence from ATTO488-DOPE, which can be present in the lower leaflet only once the water interlayer has formed, providing space for the ATTO488 at the headgroup.

Another conceivable mechanism for the lipid loss in the bilamellar regions is a transfer into vesicles. In the DC_15_PC samples, we see small vesicles with sizes below the optical resolution, as well as some larger multilamellar vesicles. The latter can be close to the bilamellar regions undergoing rearrangement into tubes, such as seen in Figure 5a–f. However, these larger vesicles before and after the rearrangement of the bilamellar regions into tubes show no increase of their lipid content (see Figure S11), ruling out significant transfer.

The membrane phase transition influences the radius of a preformed tubular structure, which we have measured by monitoring the changes in the phase profile of tubes formed from a DC_15_PC bilayer stack as the sample is cooled below its main transition temperature, *T*_m_. For this study, we used a sample with a high surface coverage (approximately 72% unilamellar, 20% bilamellar, 2% multilamellar) and a consistent overall structure (see Figure S14). This uniformity helps minimize localized tension variations in the sample resulting from differences in membrane geometry, so any observed changes in phase profile can be attributed to global effects of the phase transition acting across the whole sample.

Importantly, the phase behavior of this system is determined by the properties of a single lipid species, DC_15_PC (the effect of the 0.1 mol % fluorescently labeled DOPE is negligible). As such, tubes are expected to be entirely in the S_o_ phase after cooling. However, previous studies on SLBs on curved surfaces show that curvature on the order of tens to hundreds of nanometres can lower *T*_m_ by several degrees,^34,35^ hence the phase transition in the tubes may occur at a few degrees lower temperatures.

Figure 7 shows the integrated qDIC phase of 12 different tubes during the phase transition as the sample is cooled in 0.3 °C steps every 24 min. The temperature range over which the phase transition of planar bilayers occurs is visible in the corresponding fluorescence images (see Figure S13) by the selective partitioning of the fluorophore into the L_d_ phase coexisting with the S_o_ phases during the transition in the planar regions of the bilayer stack, and is delimited in Figure 7 by dashed lines. The integrated qDIC phase quantifies the evolution of the tube structure as the bilayer membrane changes from the L_d_ to the S_o_ phase. Within a defined lipid phase, the optical thickness of the bilayer is known, and we can convert the integrated qDIC phase to a radius, as discussed before. The right axis on Figure 7 shows the calculated radius over the phase transition assuming L_d_ phase parameters (using S_o_ phase parameters yields 21% smaller radius estimates).

**Figure 7:**
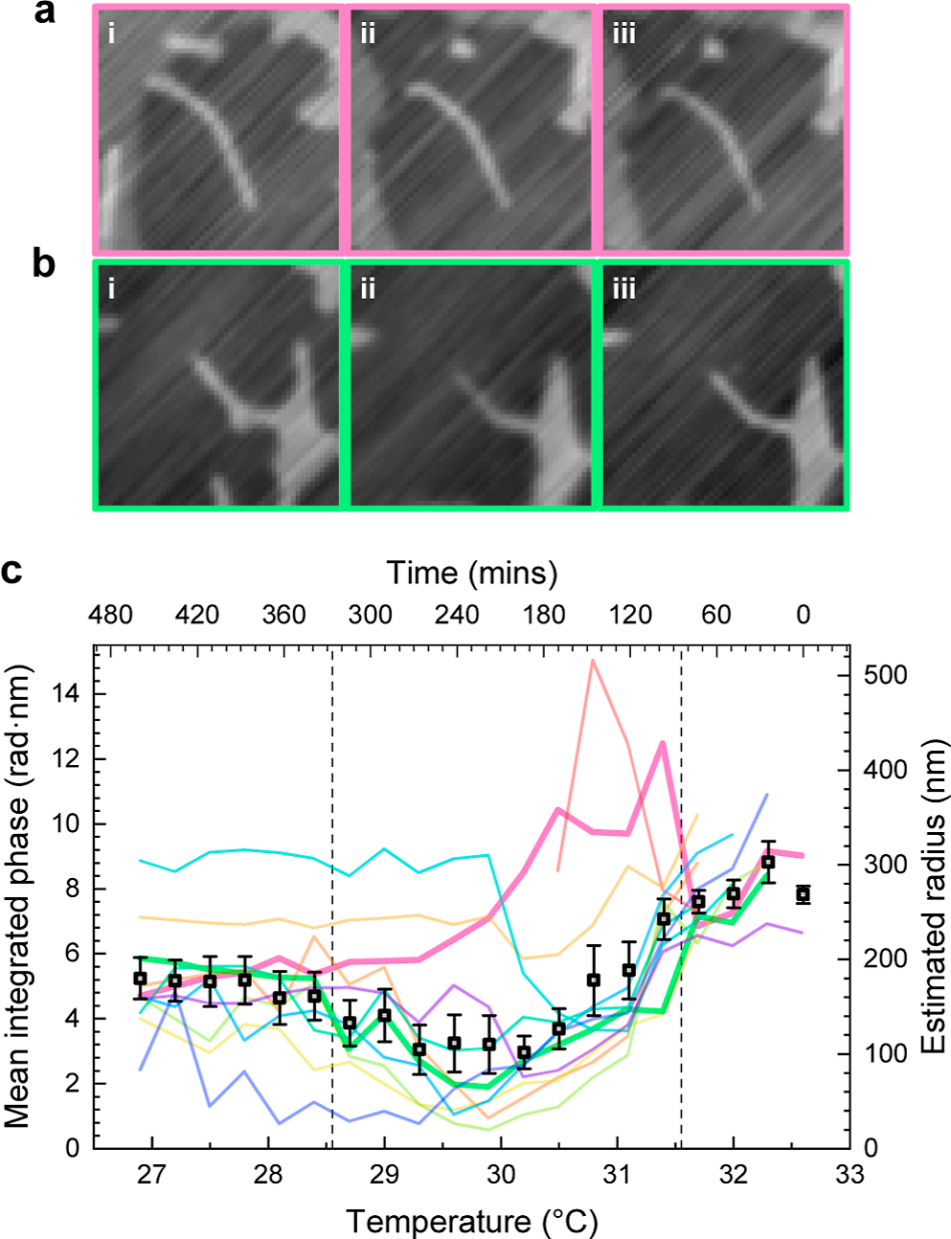
(a,b) Examples of two tubes at three different temperature points, (i) 31.7 °C, (ii) 29.3 °C, (iii) 27.5 °C. qDIC phase from −14 to 14 mrad, image size 10.8 μm square. (c) Integrated phase of 12 DC_15_PC tubes against temperature as the sample is cooled below its main phase transition temperature (the phase coexistence region in the SLB is indicated by the dashed lines). Lines corresponding to the tubes shown in a and b are highlighted in bold. The gray squares show the average for tubes remaining connected to the bilayer stack. The right axis gives the tube radius, assuming L_d_ phase parameters. The temperatures shown are target temperature of the microscope chamber. Error bars are standard deviations over the tubes. See Figure S12 for images of all tubes.

Tubes remaining connected to the bilayer stack, such as the one shown in Figure 7b, show a consistent behavior in the evolution of their integrated optical phase during cooling. The integrated qDIC phase reduces slowly until the main phase transition begins in the planar bilayer regions, then drops more sharply reaching a minimum at the center of the phase transition temperature range. This is followed by a gradual recovery that levels off once the transition is complete. Tubes that disconnect during the transition, such as the one shown in Figure 7a, represented by the pink line in Figure 7, do not follow this behavior. This indicates that the consistent behavior is driven by the common tension of structures connected to the planar bilayers during the phase transition discussed earlier.

The mean integrated phase of the tubes that remain connected to the planar bilayers is shown as gray squares in Figure 7. In the L_d_ phase, the phase area is approximately 7.8 rad nm, which corresponds to a radius of 266 nm. The radius then gradually decreases to a minimum around 107 nm as the phase transition proceeds. After the transition, an integrated phase of around 5.2 rad nm is measured, corresponding to a radius of approximately 141 nm in the S_o_ phase.

To discuss these changes in mean radius over the phase transition, we consider eq 4. The bending rigidity η scales the radius as $\sqrt{\eta }$ , and has a minimum at *T*_m_, due to the known softening near first-order phase transitions. Indeed, experiments on the structurally similar lipid DMPC have shown that η is lower by almost an order of magnitude at *T*_m_ relative to the L_d_ phase,^5^ which would lead to a three times smaller radius, consistent with the observed change between the equilibrium L_d_ phase and the minimum during phase transition.

However, η in the S_o_ phase is about five times the one of the L_d_ phase,^5^ therefore the radius would be expected to increase upon further cooling to about twice the one in the L_d_ phase, in contrast to our observation of a 30% radius reduction. This points to the influence of the membrane tension, σ, changing the radius proportional to $1/\sqrt{\sigma }$ . The about three times lower radius after cooling into the S_o_ phase than expected from the change of η would correspond to an about 9 times higher σ.

We recall that the tubes showing this characteristic shape evolution are contiguous with the unilamellar surface attached bilayer regions. As previously discussed, our understanding of the force driving tube formation is that it is more energetically favorable to pull lipids out of bilamellar regions of the film than to reduce the surface coverage of the unilamellar regions, due to the high strength of the interaction between the lower leaflet of the bilayer and the substrate. The higher lipid packing density in the S_o_ phase drives the unilamellar regions to pull lipids from elsewhere in the system to maintain their surface area upon cooling, yielding an increase of σ. This is consistent with the behavior seen in Figure 5, also requiring an increase of σ due to the surface adhered unilamellar regions and the reduction of lipid bilayer area during cooling across the phase transition.

A kinetic effect could also be contemplated, assuming that in the S_o_ phase the structure is frozen in place, not allowing lipid diffusion on the time scales observed. If the bilayer is rather described by a solid than a liquid on the relevant time scales, eq 4 is not applicable. The radius would then be smaller than expected in the S_o_ phase simply because no further lipid can be transported to the tube after freezing. However, the equilibration of the fluorophore after phase transition seen in Figure S13 indicates diffusion over a few microns within the 24 min observation time below the phase transition, which would allow sufficient transport, contradicting the assumption of a frozen structure.

## Conclusion

We have shown that lipid films with controlled lipid composition, spin-coated on a solid support, produce tubular structures, and we have quantified their radii by quantitative DIC microscopy, which is sensitive to structures with nanometre-scale lipid thicknesses below the optical diffraction limit. Tubes were analyzed in their equilibrium state using DOPC lipids which are in the liquid disordered (L_d_) phase at room temperature, and during their formation using DC_15_PC lipids which transition to a solid ordered (S_o_) phase upon cooling. Tubular networks of DOPC lipids on glass showed radii of 150–200 nm. Moreover, bilayers attached directly on glass have localized adhesion points, resulting in the formation of tubular networks, while tubes on top of lipid bilayers have little interaction with the surface and show extended straight geometries.

A complex arrangement of regions with different lamellarity is found, with surface tension created by the interaction with the glass surface dictating the tube diameter for the lipids in the liquid phase L_d_. Notably, owing to its sensitivity to birefringence, qDIC allowed us to distinguish between different tubular arrangements (e.g., concentric multilamellar versus twin unilamellar) and also reveals configurations such as folded bilayer edges and triple junctions.

Tube formation on glass is found to be driven by a depletion of lipid material from bilamellar regions of the lipid film, and a mechanism is proposed whereby lipid depletion is due to migration into unilamellar patches that are strongly adhered to the glass surface. Using DC_15_PC lipid having a phase transition from L_d_ to S_o_ phase, the characteristic sizes of tubes during cooling was studied, created by the combination of increasing surface tension and changing bending rigidity having a minimum during phase transition.

The ability to produce well-defined supported lipid tubular structures with controlled composition and geometries using this model system opens up novel experimental possibilities for dissecting lipid–lipid interactions and biophysical properties of many biological filaments existing in nature.

## Supplementary Material



## Data Availability

Information on the data underpinning the results presented here, including how to access them, can be found in the Cardiff University data catalogue at http://doi.org/10.17035/cardiff.32197758.

## Notes

Figures and sections as referenced, providing additional data and discussion (PDF)
