# Supplementary material for: Understanding the Mechanisms of Supported Lipid Membranes Reshaping into Tubular Networks Using Quantitative DIC Microscopy
Source: Langmuir. 2026 May 13;42(20):14092–103. doi: 10.1021/acs.langmuir.6c00369 (PMC13217610; doi:10.1021/acs.langmuir.6c00369)
Supplement: Supplementary file 1 [file la6c00369_si_001.pdf]

Understanding the mechanisms of supported lipid membranes reshaping into tubular networks using quantitative DIC microscopy - Supporting Information

David Regan,[1] Paola Borri,[1] and Wolfgang Langbein[2,*]

[1]*School of Biosciences, Cardiff University, Museum Avenue, Cardiff CF10 3AX, UK*

[2]*School of Physics and Astronomy, Cardiff University, The Parade, Cardiff CF24 3AA, UK*

(Dated: May 8, 2026)

CONTENTS

S1. Gradient Minimisation 2

S2. Modelling of Bilayer Tube Phase Profiles 4

S3. Regional Variation of Tube Circumference 9

S4. Water penetration between lower leaflet and substrate 10

S5. Structural Changes of the Lipid Stack During Cooling Through Phase Transition 13

S6. Measurement of Bilayer Patch Phase Area 19

S7. Shape Transformations in Tubes Cooling Across the Main Phase Transition 20

S8. Unprocessed Versions of qDIC Phase Images 28

S9. Tube Measurement Positions 31

References 36

* langbeinww@cardiff.ac.uk

S1. GRADIENT MINIMISATION

As previously described [1], several quantitative differential interference contrast (qDIC) phase images presented in the main text have been processed after Wiener deconvolution with an additional gradient minimisation step, which reduces line artefacts in the reconstructed phase images parallel to the shear direction. These line artefacts are caused by missing information orthogonal to the shear in the original differential interference contrast (DIC) images, and depend on the choice of the signal-to-noise parameter, κ, used in the Wiener filtering, which is set to 4000 in our analysis. This gradient minimisation process uses the cost function

$$E(\phi) = (\mathbf{s} \cdot \nabla\phi - \delta) + \lambda|\nabla\phi|^{\alpha} \tag{S1}$$

in which $\mathbf{s}$ is the DIC shear vector and ϕ is the reconstructed phase. The first term is the deviation between the measured differential phase image, δ, and the differential phase expected from ϕ, and serves to penalise phase reconstructions that do not reproduce the original data. The second term is the magnitude of the gradient per nanometre raised to a power α and multiplied by a weighting factor λ, and penalises gradients in the reconstructed phase. The algorithm minimises the image-averaged cost iteratively. The parameters used to generate the images shown are given in Table SI, including additional processing details where applicable. Using $\alpha = 0.5$, below unity, is suppressing weak gradients in favour of localised strong gradients, encouraging a step-like appearance of the phase, suited for layers of constant thickness such as lipid bilayers. The values of λ are chosen as small as possible to remove noise-induced stripes but not significantly affect the quantitative phase.

All line-cut analysis uses cut directions along the shear, and data without gradient minimisation. We do not use the gradient minimised data for this as it can yield less accurate line-cuts along the shear, a direction which is directly measured by qDIC.

Figure	α	λ	Iterations	Further processing
Fig. 1c	0.5	10^{-6}	2.0×10^5	N/A
Fig. 2	0.5	10^{-6}	1.0×10^4	N/A
Fig. 3a, Fig. S26	0.5	10^{-6}	1.0×10^4	Subtracted linear background
Fig. S7, Fig. S8a,b	0.5	10^{-7}	1.0×10^5	N/A
Fig. S9a	0.5	10^{-7}	1.0×10^4	Gradient minimisation was applied to a crop from the full image for speed. This introduced of edge artefacts in the minimisation. These edge artefacts were removed as follows. First, the difference between the original phase reconstruction and the new gradient minimised reconstruction was taken to separate the artefacts from real image structure. Next, this difference was fitted with a fourth order polynomial to fit just the new artefacts. Finally this polynomial was subtracted from the gradient minimised image.
Fig. S9b-c	0.5	10^{-7}	1.0×10^4	N/A

TABLE SI. Parameters used in the qDIC global minimisation results shown.

S2. MODELLING OF BILAYER TUBE PHASE PROFILES

To support the interpretation that the branched structures in the DIC images are tubes, we presented in Fig. 3 simulations of the expected phase profile of different cross-sectional shapes as follows: unilamellar and bilamellar tubes with circular cross-sections; stacks of two unconnected flat bilayers; and an intermediate "flattened tube" configuration, in which the radius of curvature at the edges of the tube was fixed at 64.8 nm. These simulations were generated in MATLAB R2021a.

Simulated phase profiles were produced as projection of the cross-section lengths per lateral distance, at a resolution of 0.01 nm. In the simulations, we made some simplifying assumptions about the cross-section. For bilamellar tubes, we assumed the two lamella have zero separation. For the case of the flattened tube, we assumed that the tube was only curved at the edges, and that the bilayer was otherwise flat. For circular regions of the tube cross-sections, projected lengths were calculated analytically as function of distance x from their center to be

$$l(x) = 2\,\mathrm{Re}\left(\sqrt{R_{\mathrm{out}}^2 - x^2} - \sqrt{(R_{\mathrm{out}} - Nd)^2 - x^2}\right), \tag{S2}$$

where R_{out} is the outer radius of the tube, N is lamellarity of the tube, and d is the bilayer thickness (set to be 4.52 nm in our simulation based on our earlier work [1]). For the flat regions of the cross section, the projected length l is simply $2Nd$. Lengths were then converted to phases ϕ using

$$\phi = \frac{2\pi l(n_{\mathrm{bilayer}} - n_{\mathrm{medium}})}{\lambda}, \tag{S3}$$

where here λ is the illumination wavelength (set to 550 nm to match the experimental conditions), and n_{bilayer} and n_{medium} are the refractive indices of the bilayer (1.445) and surrounding medium (1.3341) respectively. The simulation assumes the same refractive index inside and outside the tube.

The simulated phase profiles were then convolved with a sech^2 function to model the point spread function of the microscope, consistent with the fit function Eq.(3). The width parameter of the sech^2 function (0.567 of its full width at half-maximum) was chosen to be $w_0 = 262.6$ nm based on the width of the tanh fits to bilayer edges in our previous work. [1]. The profiles were then downsampled using MATLAB's built-in resample function to match

the spatial resolution of our camera (216.1 nm/pixel). Finally, the resultant profiles were fitted using Eq.(3), fixing f to zero since the simulated data contains no birefringence. All fitting was carried out using built-in fitting tools in MATLAB using the same procedure as for the measured line profiles.

In addition to the profiles shown in Fig. 3f-g, we simulated a number of other cross-section shapes. These include bilamellar circular cross-section tubes in which the inner tube has 80%, 60%, and 40% the radius of the outer tube, and bilamellar flattened tubes with zero lamellar separation. The results for all considered cross-section shapes are shown in Fig. S1.

We supported our interpretation that the tubes are slightly flattened relative to a perfectly circular cross-section using the measured phase step over the bilayer tubes due to bilayer birefringence, as shown in Fig. 3e. We estimated the expected height of the phase step for a tube with circular cross-section as follows. First, we consider the phase difference at each point $\mathbf{r}$ in the image neglecting birefringence, given by

$$\delta'(\mathbf{r}) = \phi\left(\mathbf{r} + \frac{\mathbf{s}}{2}\right) - \phi\left(\mathbf{r} - \frac{\mathbf{s}}{2}\right), \tag{S4}$$

where $\mathbf{s}$ is the DIC shear, and $\phi(\mathbf{r})$ is phase created by the optical thickness of the tubular structure at a given point on the sample. The two terms represent two orthogonal polarisations along and across the shear, which are spatially separated on the sample by the shear. In the phase difference including birefringence δ, the polarisation orthogonal to the tubular structure, and thus along the shear, experiences a mixture of ordinary and extraordinary refractive index, resulting in a scaling factor $(1+\alpha)$ on the phase for the ordinary refractive index experienced by the polarisation along the tubular structure, so that

$$\delta(\mathbf{r}) = \phi\left(\mathbf{r} + \frac{\mathbf{s}}{2}\right)(1+\alpha) - \phi\left(\mathbf{r} - \frac{\mathbf{s}}{2}\right) = \delta' + \phi\left(\mathbf{r} + \frac{\mathbf{s}}{2}\right)\alpha\,. \tag{S5}$$

We convert the phase differences to phase (ϕ and ϕ') by integration along the shear direction,

$$\phi = \frac{1}{s}\int \delta d\mathbf{r} = \phi' + \frac{1}{s}\int \phi\left(\mathbf{r} + \frac{\mathbf{s}}{2}\right)\alpha d\mathbf{r}\,, \tag{S6}$$

where $s = |\mathbf{s}|$ is the shear distance. The birefringence phase of the tube, ϕ_{b}, is thus given by

$$\phi_{\mathrm{b}} = \phi - \phi' = \frac{\alpha}{s}\int \phi d\mathbf{r}\,. \tag{S7}$$

Since the birefringence is a weak effect (i.e. $\alpha \ll 1$), it can be reasonably assumed that $\phi \approx \phi'$, and we can use the optical thickness of a lipid bilayer tube calculated for the case

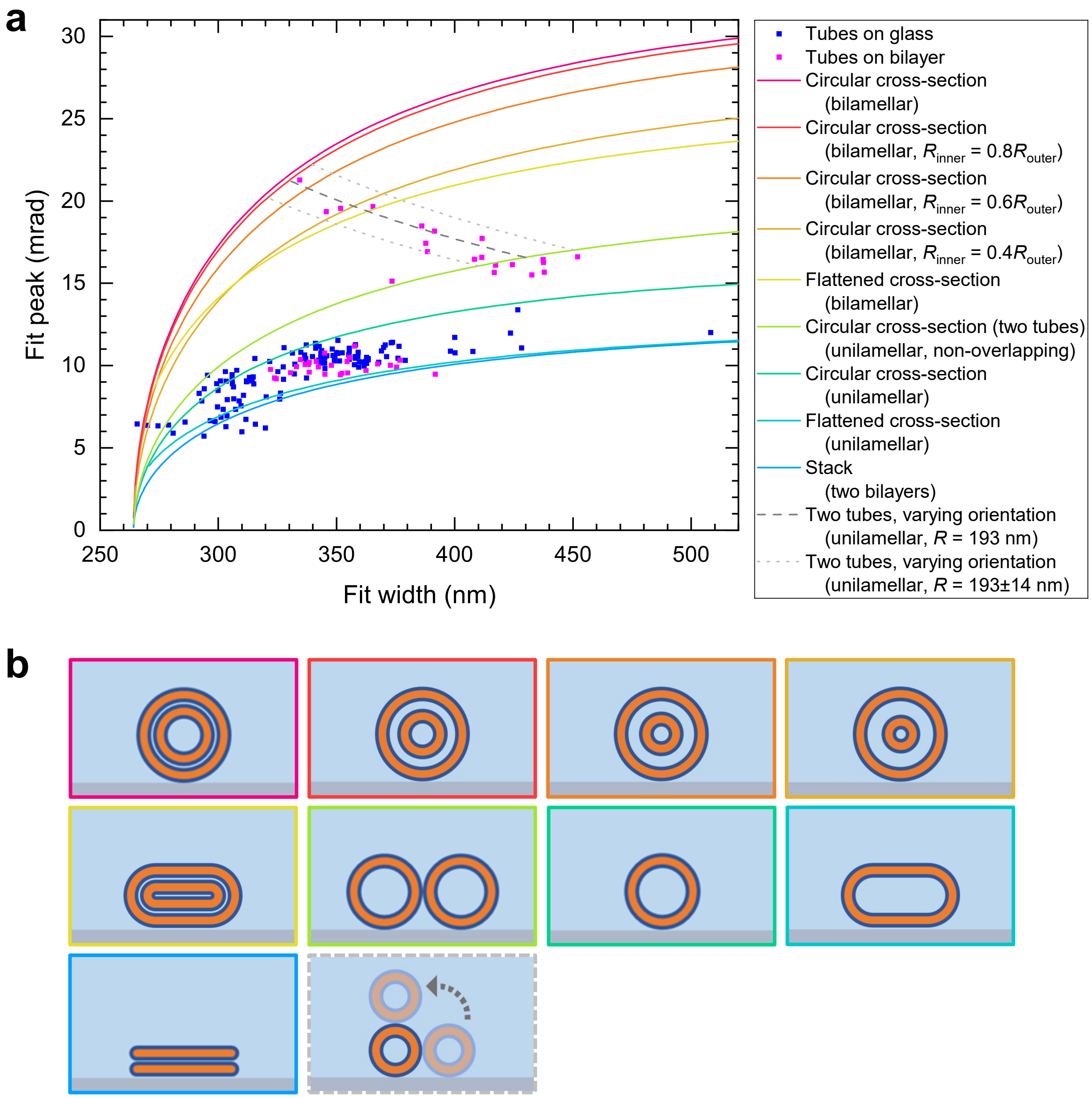

FIG. S1. Supplement to Fig. 3f showing additional cross-section shapes. a) Fitted peak amplitude a against width c, from the fitting to the 1,2-dioleoyl-sn-glycero-3-phosphocholine (DOPC) tube phase profiles, with simulated curves for different cross-section shapes shown as coloured lines. b) Illustration of the different cross-section shapes simulated in a), with corresponding frame colour.

without birefringence to estimate the birefringence phase as

$$\phi_\mathrm{b} \approx \frac{\alpha}{s} \int \phi' d\mathbf{r}\,. \tag{S8}$$

The value of α in Eq.(S8) is dependent on the shape of the tube cross-section. The

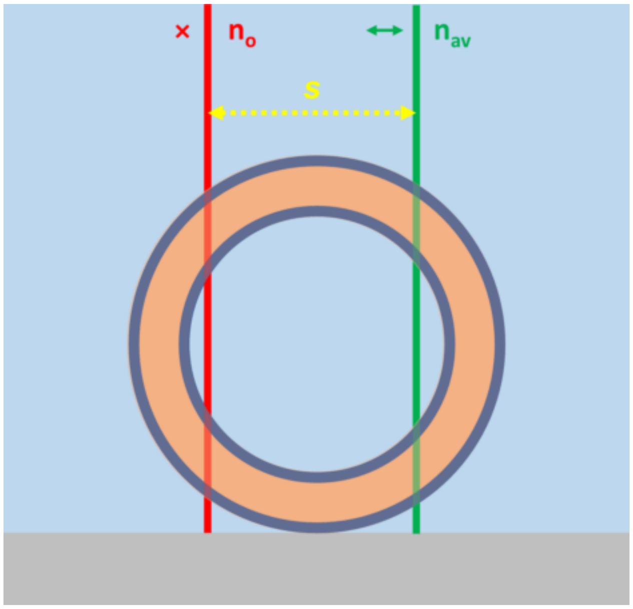

FIG. S2. Illustration of the different refractive indices experienced by the two DIC polarisations (red, out-of-plane polarisation; green, in-plane polarisation), where **s** is the DIC shear distance.

polarisation along the tube experiences only the ordinary refractive index, n_o, while the other experiences a geometry-dependent average (n_av) of ordinary and extraordinary (n_e) indices, as illustrated in Fig. S2. In case of a circular cross-section, this average refractive index is given by

$$n_\mathrm{av} = \frac{n_\mathrm{o} + n_\mathrm{e}}{2}\,. \tag{S9}$$

As the change in the optical thickness ϕ depends on the difference between the refractive index of the lipid and that of the phosphate-buffered saline (PBS) medium, we have

$$\alpha + 1 = \frac{n_\mathrm{av} - n_\mathrm{PBS}}{n_\mathrm{o} - n_\mathrm{PBS}}\,, \tag{S10}$$

and introducing $n'_\mathrm{e,o} = n_\mathrm{e,o} - n_\mathrm{PBS}$ we find for a circular cross-section

$$\alpha = \frac{n'_\mathrm{e}}{2n'_\mathrm{o}} - \frac{1}{2}\,. \tag{S11}$$

For 1,2-dioleoyl-sn-glycero-3-phosphocholine (DOPC), $n_\mathrm{o} = 1.445$ and $n_\mathrm{e} = 1.460$ [2], we find $\alpha = 6.8\%$. In the fit function, the step is $2af$ and the phase integral $2ac$, so that Eq.(S8) results in a birefringence factor $f = \alpha c/s$. For tubes with a circular cross-section of circumference 1112 nm (radius 177 nm), using an optical thickness of a single bilayer of 5.73 mrad [1], Eq.(S8) predicts a phase step of 1.81 mrad. In contrast, we find for the

measured data an average phase step $2af$ (see Eq.(3)) of 0.60 mrad, indicating that the tubes are on average not circular but flattened. For the configuration in Fig. 3g, which assumes semi-circle edges of radius 64.8 nm and no other curvature, the birefringence is given by the two edge regions only, which together are forming a circular tube of this smaller radius, reducing the predicted phase step to 0.64 mrad, consistent with the measured average value.

S3. REGIONAL VARIATION OF TUBE CIRCUMFERENCE

In the main text, we discuss that in the liquid phase L_d the tube circumference is controlled by the tension in the contiguous bilayer domain the tube is connected to, and gave an example in Fig. 2. In Fig. S3 we show the full field of view of the data as qDIC phase image without gradient minimisation. Lines are overlaid indicating the positions of measured profiles across tubes, with a colour encoding the resulting tube circumference. We see that connected tube networks have generally a similar circumference. The region on the bottom left shows the smallest radii, indicating the highest tension.

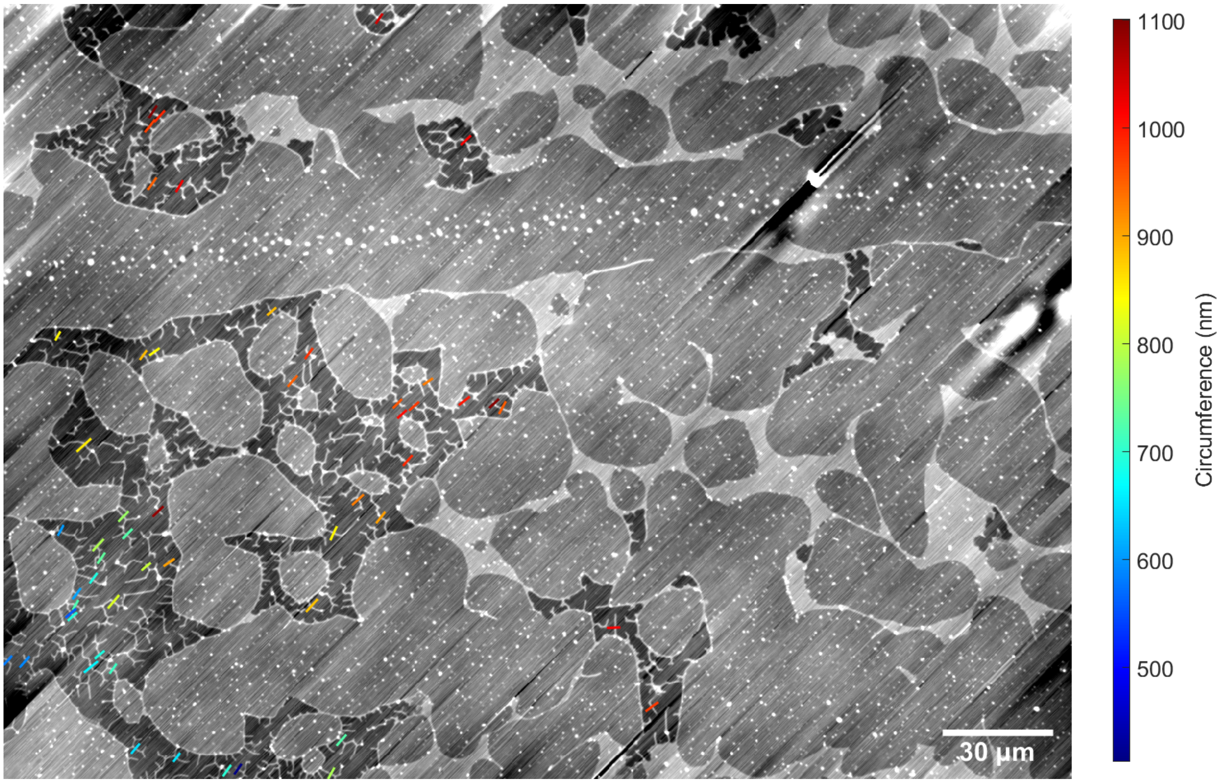

FIG. S3. Full field of view of the qDIC phase image containing the region shown in Fig. 1 and Fig. 2, scaled from -10 mrad to 10 mrad. Lines are overlaid indicating the positions of measured profiles across tubes, with a colour encoding the resulting tube circumference on a scale as indicated.

S4. WATER PENETRATION BETWEEN LOWER LEAFLET AND SUBSTRATE

We showed in our previous work that strong bilayer–surface interactions on samples prepared by spin-coating occur, as seen by a stretching of the upper leaflet of the surface-attached bilayer, evidenced by comparing unilamellar and bilamellar regions [1]. These lipid bilayer stacks are formed by hydration of a dry lipid film, and thus the lipid-surface interaction is established in the absence of water. Upon hydration, water apparently cannot penetrate between the substrate and the adhered lipid headgroups. This is different from samples formed by SUVs rupturing onto a polar glass surface, where the polar headgroups are already coordinated with water when arriving at the surface, which leads to a uniform hydration layer separating the first bilayer from the substrate.

In the main text we propose that this adhesion results in a conservation of surface area of lower leaflets of unilamellar regions, and that upon cooling from the L_d to the S_o phase, lipid is moving to the upper leaflet of the surface attached bilayer, as required by the higher areal density of the S_o phase. In bilamellar regions instead, the tension in the upper bilayer wrapping around to the lower bilayer at the edges can gradually pull off the lower bilayer from the surface allowing for coordination of water with headgroups and substrate. This separates the bilayer from the surface, except at a small number of contact points, which could be formed by holes in the glass affecting the edge geometry.

We have indirect evidence of a water hydration layer forming adjacent to a region of direct contact between the bilayer and the glass surface for supported lipid bilayers (SLBs) of DOPC formed in 2 mM Tris buffer solution (instead of the PBS solution used in our experiments otherwise). The corresponding fluorescence and qDIC image data is given in Fig. S4 and shows a border between two regions with a step change in fluorescence (magenta arrows in Fig. S4a), without a corresponding feature in qDIC. The line traces shown in Fig. S4c support this observation.

We suggest that the region with higher fluorescence intensity has a water hydration layer between the first bilayer and the surface, allowing the fluorescent ATTO488-DOPE to be present in the lower leaflet, since the water layer provides space for the ATTO488 attached to the DOPE headgroup. Conversely, the region with lower fluorescence intensity has the bilayer directly bound to the glass, so that ATTO488-DOPE is excluded since the dye sterically hinders the binding of the lipid head-group to the surface.

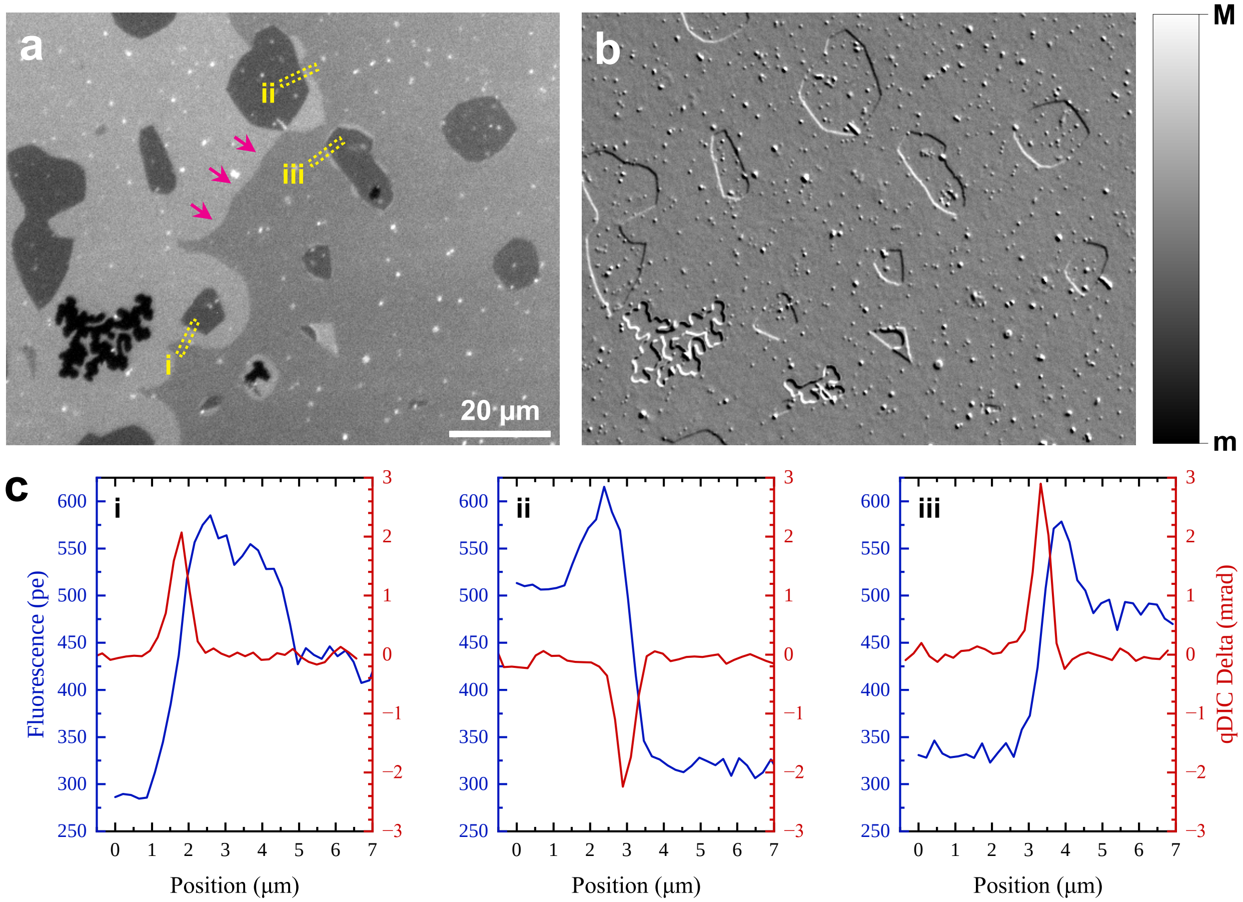

FIG. S4. A mostly bilamellar region of a DOPC supported lipid bilayer (SLB) containing unilamellar patches, shown in a) fluorescence ($m = 0$ to $M = 920$ photoelectrons), b) qDIC δ ($m = -3$ to $M = 3$ mrad). A borderline with a step in fluorescence (see the magenta arrows) is observed with no corresponding structure visible in the qDIC. The change in intensity over the yellow lines in a) are shown in c) for fluorescence (blue) and qDIC phase (red).

Notably, upon quantitative analysis of the fluorescence intensity, we find that the fluorescence step is a reduction by about 25%, as expected for a bilamellar region when removing the fluorescence from one leaflet. In bilayer stacks formed in PBS, we have not observed such a behaviour, and we suggest that the formation of the hydration layer occurs in this case on timescales longer than the experiment. This difference can be rationalised considering the chemical properties of Tris, which can penetrate into the bilayer and can form hydrogen bonds to the glass surface, competing with the lipids.

The most obvious alternative explanation of the fluorescence contrast is that this feature is simply caused by an additional bilayer. However, even a single bilayer would produce

an optical thickness of approximately 5 mrad [1], which would be readily detectable using qDIC, contrary to our observations. Another explanation that may be suggested would be that the step arises from a phase boundary between L_d and fluorophore-excluding L_o phases. However, room-temperature DOPC SLB are not expected to phase separate, and we have previously demonstrated that such phase boundaries are directly visible in qDIC, having an optical thickness step of around 1.5 mrad[1]. Conversely, the formation of a hydration layer naturally leads to fluorescence contrast via steric exclusion of the fluorophore, while producing no optical thickness boundary visible in qDIC, in agreement with our observations.

S5. STRUCTURAL CHANGES OF THE LIPID STACK DURING COOLING THROUGH PHASE TRANSITION

We note that in some experiments on 1,2-dipentadecanoyl-sn-glycero-3-phosphocholine ($DC_{15}PC$) bilayers, for example in Fig. 6, following conversion of the bilamellar regions of the lipid film to tubular networks, we see the detachment of the unilamellar regions from the surface. We show the progression of this behaviour in qDIC phase images in Fig. S5 (see Fig. S6 for corresponding fluorescence images), where between a) and b), minimal change in the boundaries of the unilamellar region occurs, while starting from c), progressive loss of

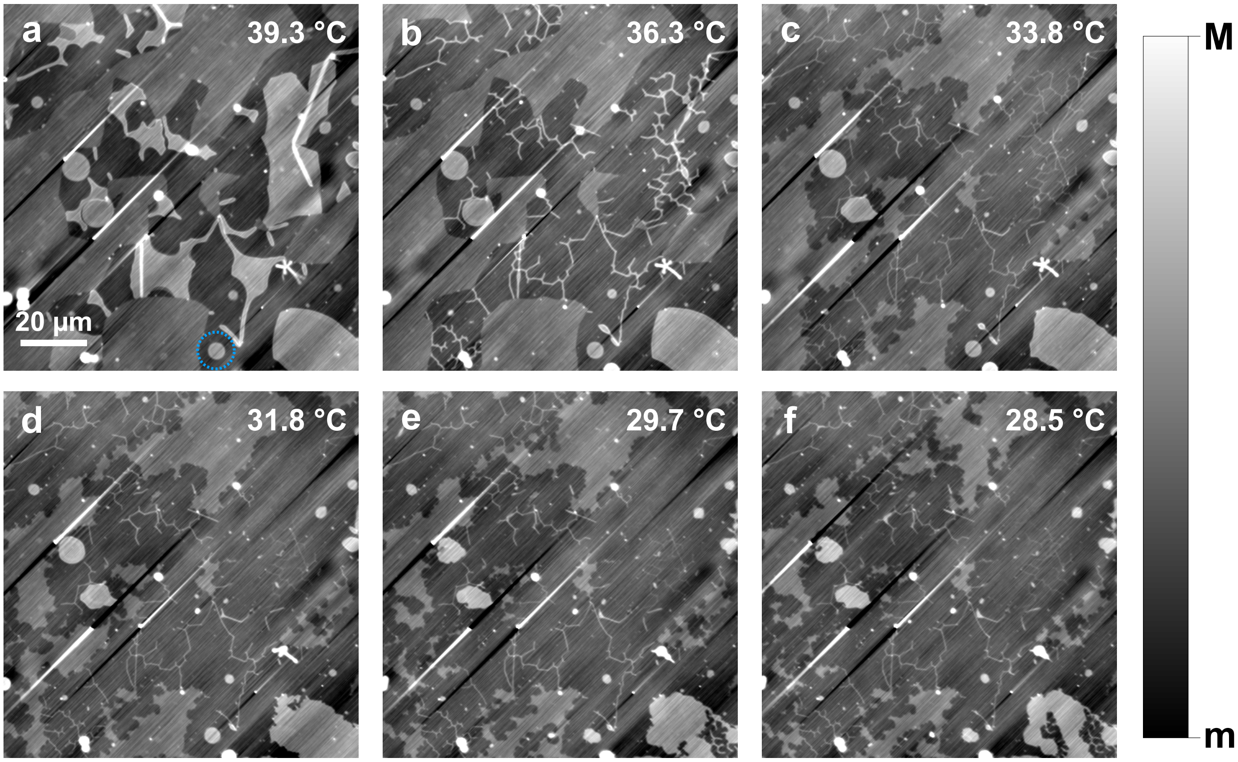

FIG. S5. qDIC phase images of shape transformations in a 1,2-dipentadecanoyl-sn-glycero-3-phosphocholine ($DC_{15}PC$) bilayer stack during cooling, showing the depletion of lipids from the bilamellar regions leading to the formation of membrane tubes, followed by the gradual loss of area from the unilamellar regions. Image is scaled from $m = -3$ mrad to $M = 20$ mrad. The strong black/white stripes along the shear are not visible in fluorescence (see Fig. S6), and are therefore not due to lipids. They are attributed to unknown nanoparticles which are birefringent due to shape and/or material, resulting in a step response in the integrated qDIC phase.[3]

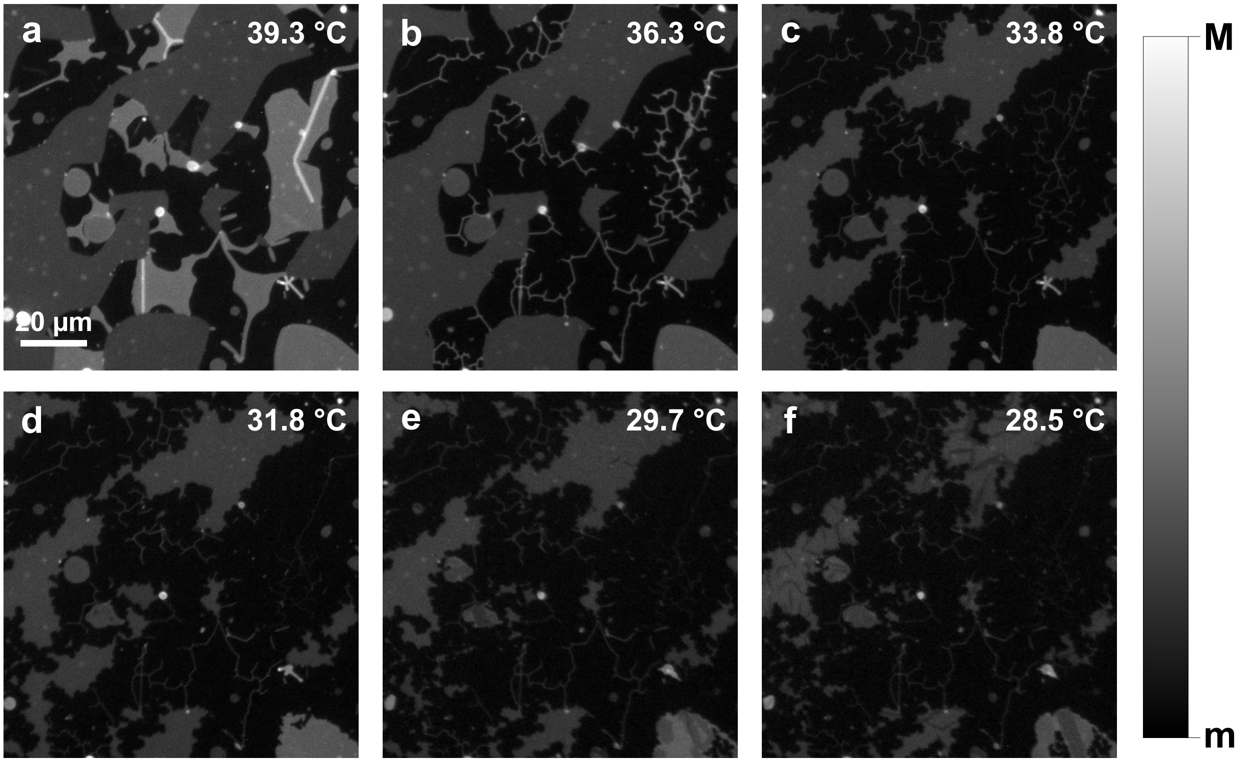

FIG. S6. Fluorescence images corresponding to Fig. S5. Images are scaled from $m = 0$ to $M = 210$ counts.

the unilamellar regions occurs as no more material can be extracted from the tubes. This is interpreted as transport to more strongly attached unilamellar regions or tubular networks outside the field of view, contiguous with the bilayer observed. The hypothesis is that as the available bilayer surface area decreases during cool down across the phase transition, due to the increase in molecular surface density towards the S_o phase, the bilayer surface tension increases until the weakest surface attachment region detaches, leading to a stepwise remodelling of the shape. Notably, we observe at the same time that roundish bilayer regions which are separated from other bilayers (such as the small patch indicated in Fig. S5a by the blue circle) keep their shape until the transition to the S_o phase results in polygonal shapes due to the softening of the bending rigidity at small radii, so that structures with sharp bends combined with flat regions have lowest bending energies, as also observed in GUVs [4].

Connected tubular networks show a synchronous stepwise decrease of circumference, evidence for a surface tension controlled radius. This can be seen in Fig. S7 where tubes in

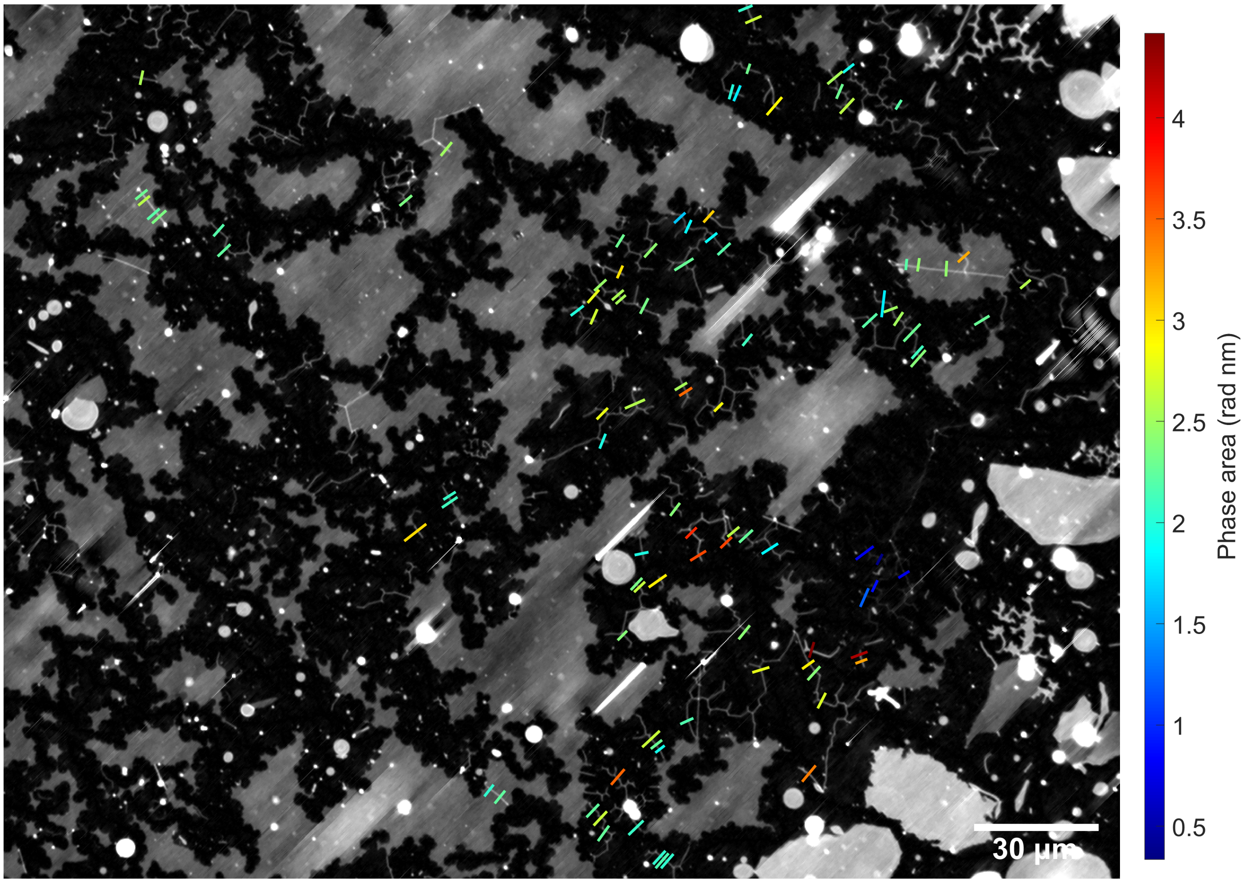

FIG. S7. Minimised qDIC phase image containing the region shown in Fig. S5 at 32.3 °C, scaled from 0 mrad to 16 mrad. Lines show measured profiles across tubes, with a colour encoding the resulting tube phase area on a scale as indicated.

different networks have different phase areas. Fig. S8 shows the phase area for three example networks during cooldown. Note that tubes in the networks indicated by the squares and circles (highlighted in red and green respectively in Fig. S8a,b) initially show a common behaviour during cooling, since they are attached to the same unilamellar region and thus experience similar tension, but become independent once the network on the right becomes disconnected from the unilamellar bilayer regions and the tensions in each network become different.

In the $DC_{15}PC$ sample shown in Fig. 5, we also see examples of tubes forming on top of other bilayers. In Fig. S9a a trilamellar region of the lipid stack in which the top two bilayers are folded over into a long, thin proto-tube structure. The radius of this structure is initially very large, well above the optical resolution of our imaging system (illustrated in

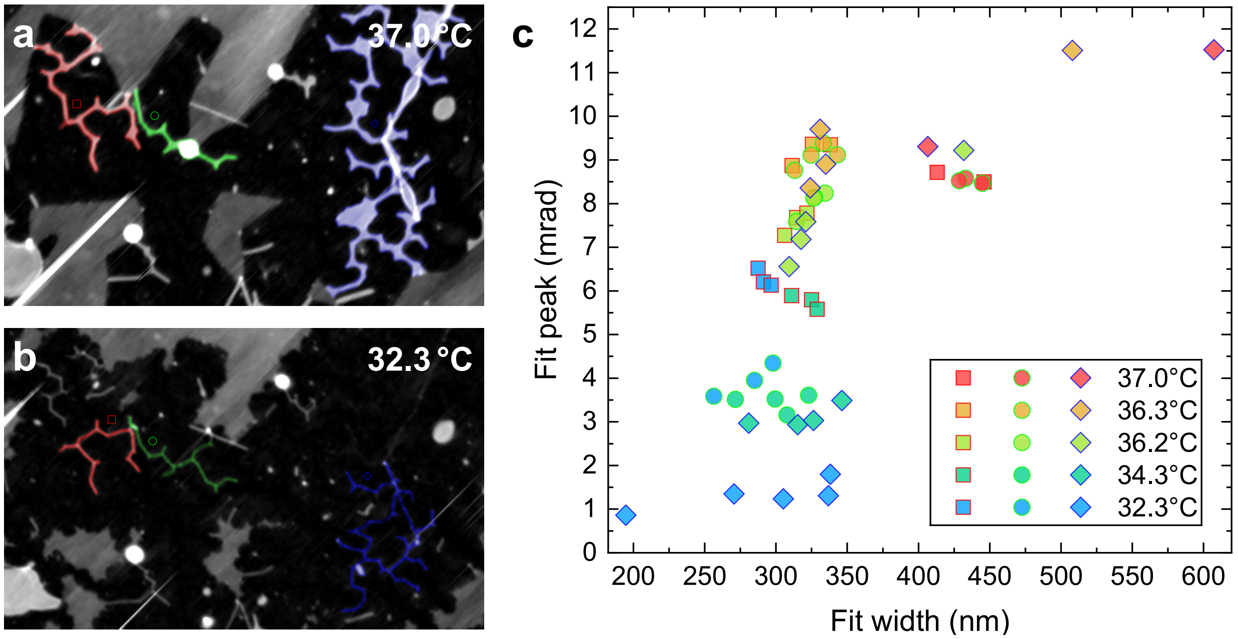

FIG. S8. a,b) Minimised qDIC phase image with the three measured tubular networks highlighted in red (squares), green (circles), and blue (diamonds), scaled from 0 mrad to 16 mrad. Image width is 75.6 µm. c) Fit parameters for measurements of tubes in each of the three networks.

Fig. S9d,i), but gradually decreases over time, eventually adopting a tube arrangement. This decrease indicates that it is subject to the same lipid depletion that affects the bilamellar patches (see Fig. 5), suggesting that this tube must be contiguous with the unilamellar regions of the lipid film. This is consistent with our observations on the DOPC tubes where the common tension between the tubes on glass and those formed on top of other bilayers indicated a direct connection. We note that some other higher lamellarity structures visible in Fig. S9a-c, such as the circular patch next the tube (cross-section illustrated in Fig. S9d,ii) do not change size over time, indicating they are isolated from the unilamellar regions of the stack. These are assumed to be situated below the unilamellar sheet to provide the observed position stability.

We suggest that the movement of lipids from the tube to the underlying unilamellar region occurs at triple junction structures similar to those we propose exist at the boundary between unilamellar and bilamellar regions (Fig. S9d,iii). In figure Fig. S9d,iv we illustrate what the interface between the tube and the bilamellar region may look like. We also illustrate the cross-section over a trilamellar patch in the top left corner of Fig. S9a which

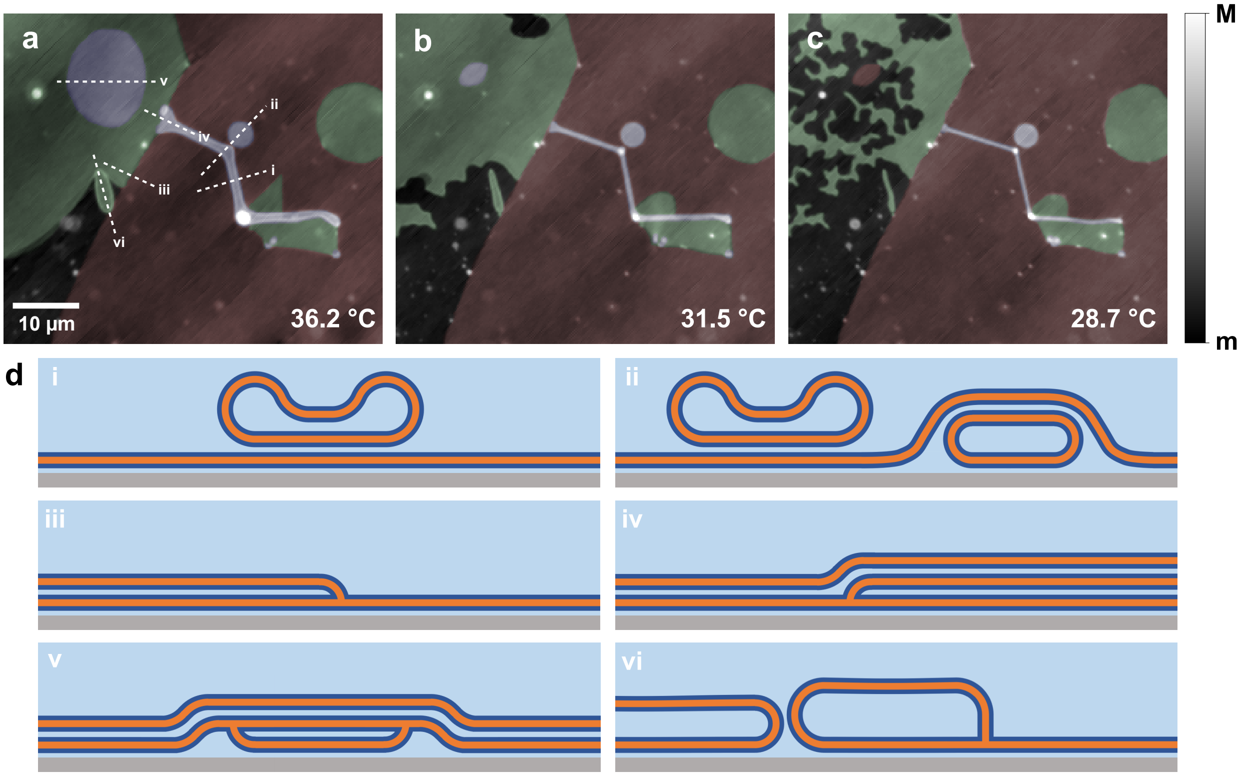

FIG. S9. a-c) Minimised qDIC phase images showing the formation of tubes on top of a unilamellar region of a $DC_{15}PC$ lipid film during cooling below the main phase transition temperature T_m, scaled from m = -8 mrad to M = 24 mrad. The lamellarity of the sample is indicated by a colour overlay, highlighting single bilayer regions (red), double bilayer regions (green), and regions of higher lamellarity (blue). d) Illustrations of hypothesised cross-sections along the different lines marked in a).

is consumed as the bilamellar surrounding region contracts (Fig. S9d,v); we suggest that lipid movement again occurs via triple junction structures linking the bilayer in contact with the surface to the bilayer above, and that these triple junction structures detach as the two bilayers on top recede, leaving behind a small unilamellar patch. An elongated vesicle attached to the unilamellar regions which also exhibits lipid loss gradually adopting a more tube-like structure is shown in Fig. S9d,vi.

In the same sample, we also observed instances of large isolated bilamellar patches undergoing shape rearrangement into large vesicles. An example of this is shown in fluorescence in Fig. S10. Notably a tubular network remains on the surface, consistent with the idea of surface pinning points discussed in the main text. The vesicle away from the surface forms at

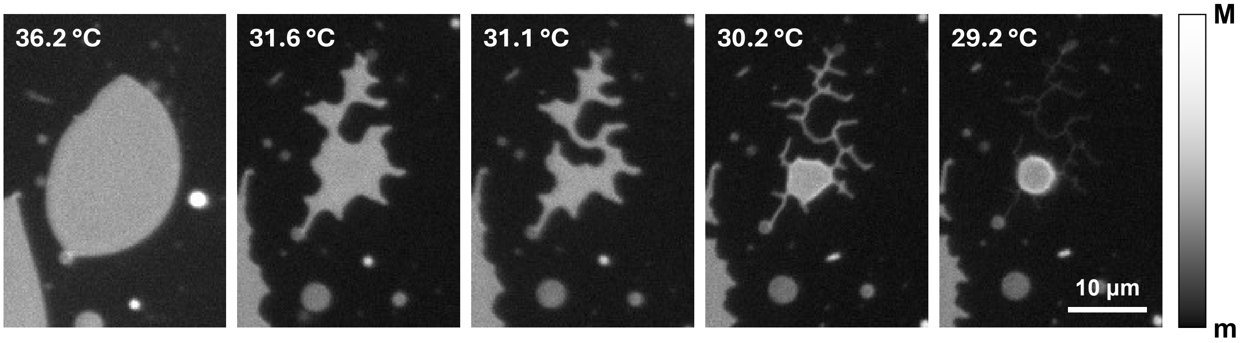

FIG. S10. Fluorescence images showing a shape transformation from a bilamellar patch on the glass surface to a large vesicle in the $DC_{15}PC$ sample shown in Fig. 5. Image is scaled from $m = 40$ to $M = 300$ counts.

29.2 °C, and has an polygonal shape typical of a solid phase S_o. The tubes at the same time get very thin (approximately 33 nm radius), an indication that the surface tension increased to large values during the freezing of the bilayer. The role of the internal water volume is relevant in this closed bilamellar structure. As the internal volume is conserved, the tube volume is reduced and a vesicle with a few kinks to minimise the bending energy is formed. We can also see a small disconnected round vesicle transforming into a brick-like structure, likely due to the same mechanism.

To determine whether such large vesicles were the cause of lipid depletion from the supported lipid bilayer stack, we measured the phase profile through the multilamellar vesicle at the centre of the field of view in Fig. 5a-f (the position of which is indicted by the dagger in Fig. 5a) at two temperatures. The results are shown Fig. S11. It can be seen that the phase profile for the two temperature points is similar. The difference in height between the two peaks on either side of the phase profile is the result of the birefringence of the lipid bilayer. Assuming cylindrical in-plane symmetry to estimate the total phase area from the phase profile through the vesicle center, we find at 36.2 °C a phase area of 0.89 rad μm^2, which corresponds to a total lipid bilayer area of 187 μm^2 using a L_d bilayer optical thickness of 4.756 mrad. Considering the vesicle radius of 1.87 μm, this corresponds to a lamellarity of 4. After cooling to 28.7 °C, the phase area was 0.79 rad μm^2, corresponding to a total lipid bilayer area of 150 μm^2, using a S_o bilayer optical thickness of 5.279 mrad. The L_d and S_o optical thicknesses are taken from fits to $DC_{15}PC$ bilayer data of the present work. The difference in phase area is within the uncertainty of the measurement.

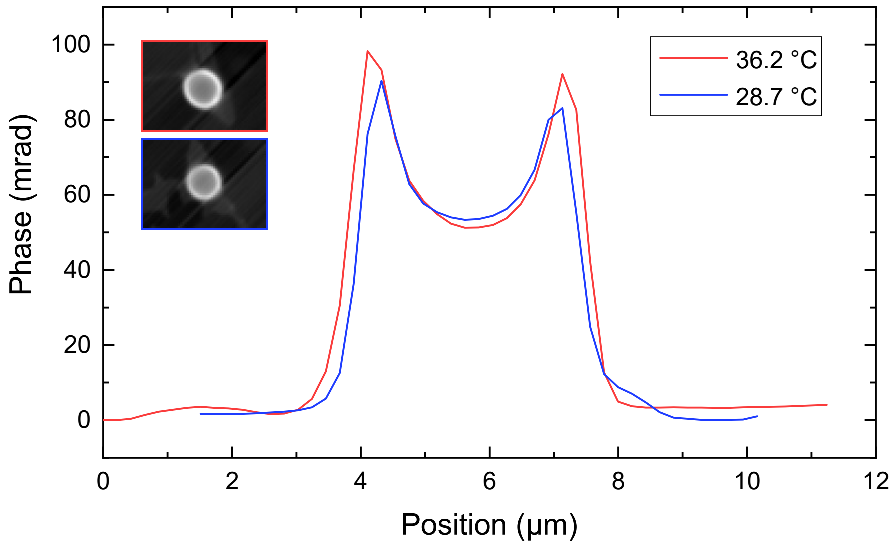

FIG. S11. Phase profile through a multilamellar vesicle (a 12.1 μm wide region is shown as an inset) adhered to the supported lipid bilayer stack shown in Fig. 5a-f.

S6. MEASUREMENT OF BILAYER PATCH PHASE AREA

In the main text we present measurements of the phase area of two independent $DC_{15}PC$ bilayer patches (indicated by the asterisks in Fig. 5b) at temperature points above and below the nominal phase transition temperature. In order to measure the phase area, first, the patches were cropped out of the larger field of view, and a binary mask was created manually to distinguish the patch from the background region. Then, a second-order polynomial is fitted to the region around the patch in order to obtain a fit to the background phase. This background is then subtracted from the data, which is then multiplied by the mask to set regions outside the patch to zero. The data is then summed and multiplied by the square of the pixel size in microns Fitting is carried out in MATLAB using the built-in fit function.

S7. SHAPE TRANSFORMATIONS IN TUBES COOLING ACROSS THE MAIN PHASE TRANSITION

In the main text Fig. 7 the effect of the main phase transition on the integrated phase on twelve different tubes is shown. The integrated phase measured for the each of these twelve tubes individually is given in Fig. S12a, and their spatial shapes near the start of cooling are shown in Fig. S12b. For this data set, in order to minimise photobleaching of the fluorescent

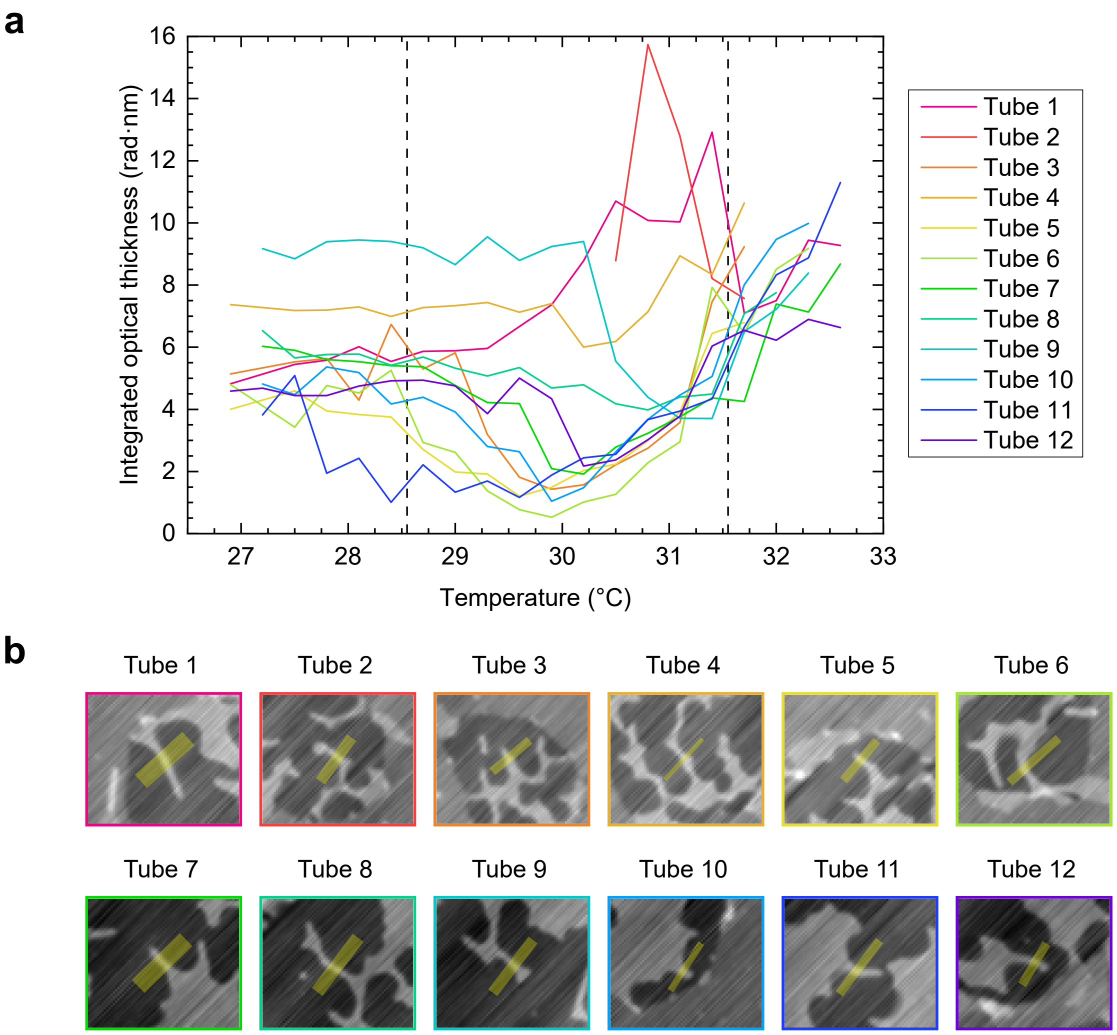

FIG. S12. a) Integrated phase against temperature for the twelve tubes in Fig. 7. b) qDIC phase images of each of the twelve tubes in the fluid phase, on a scale from −14 to 14 mrad. Tubes 1 to 6, and 12, are shown at the temperature corresponding to the starting point (highest temperature) of their respective time traces in a), while tubes 7-11 are shown one point (0.3 °C) below the starting point.

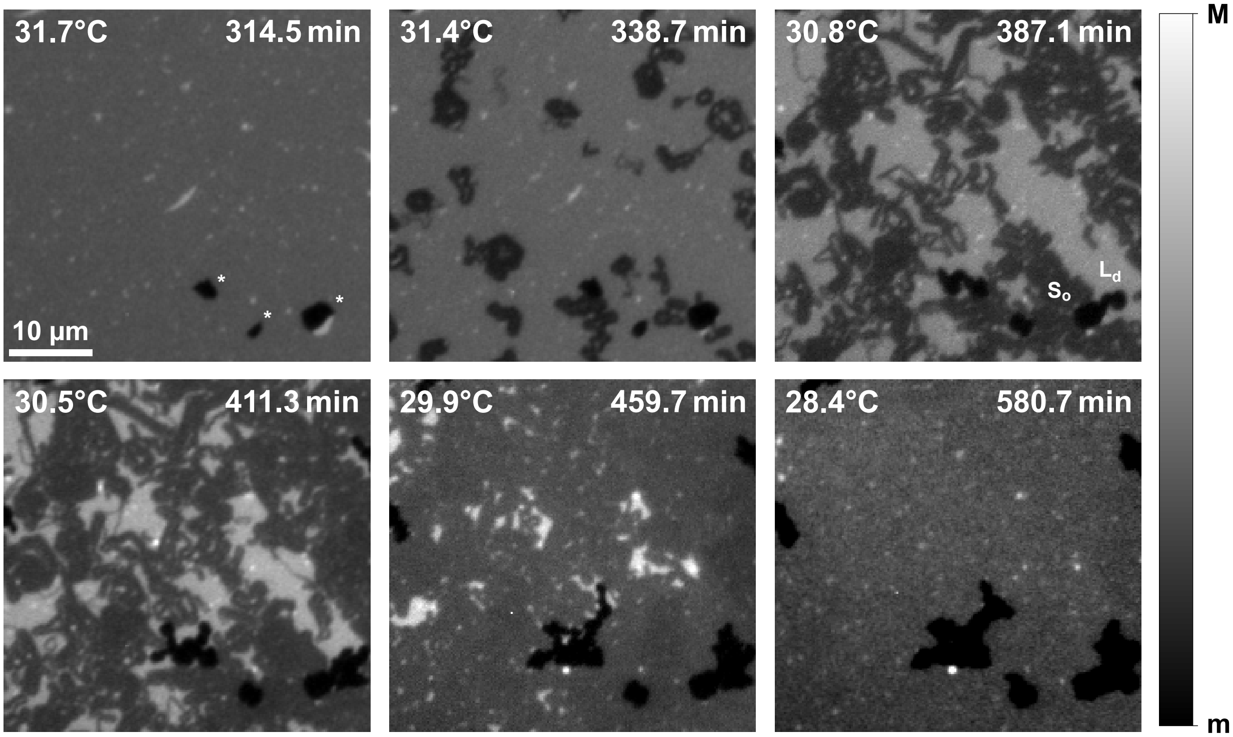

FIG. S13. Fluorescence images showing the gradual phase transition in a planar unilamellar bilayer of $DC_{15}PC$ during cooldown through the L_d to S_o phase transition, scaled from $m = 0$ to $M = 800$ counts. The fluorophore-labelled DOPE is first excluded from frozen (S_o) regions of $DC_{15}PC$, resulting in L_d patches of higher fluorescence, until after completion of the phase transition the fluorophore-labelled DOPE redistributes across the bilayer of S_o phase during approximately 30 minutes. Holes in the bilayer are indicated by asterisks in the first image to distinguish them from fluorophore excluding domains.

lipids during imaging, the DIC images were acquired using a Semrock Brightline FF01-607/36-25 bandpass filter instead of the Nikon GIF filter, resulting in a centre wavelength of 607 nm instead of 550 nm.

Regions of the fluorescence images of planar unilamellar regions in the same bilayer stack at different temperatures during the phase transition are shown in Fig. S13. The edges of phase coexistence regions (28.55 °C and 31.55 °C) indicated in Fig. S12b and Fig. 7 are determined based on the fluorescence images. The midpoint between the last measured temperature in which the sample appears to be in a single phase L_d (31.7 °C) and the first temperature point in which fluorophore excluding domains appear (31.4 °C) is taken as start of the phase transition. Likewise, the midpoint between the last measured temperature in

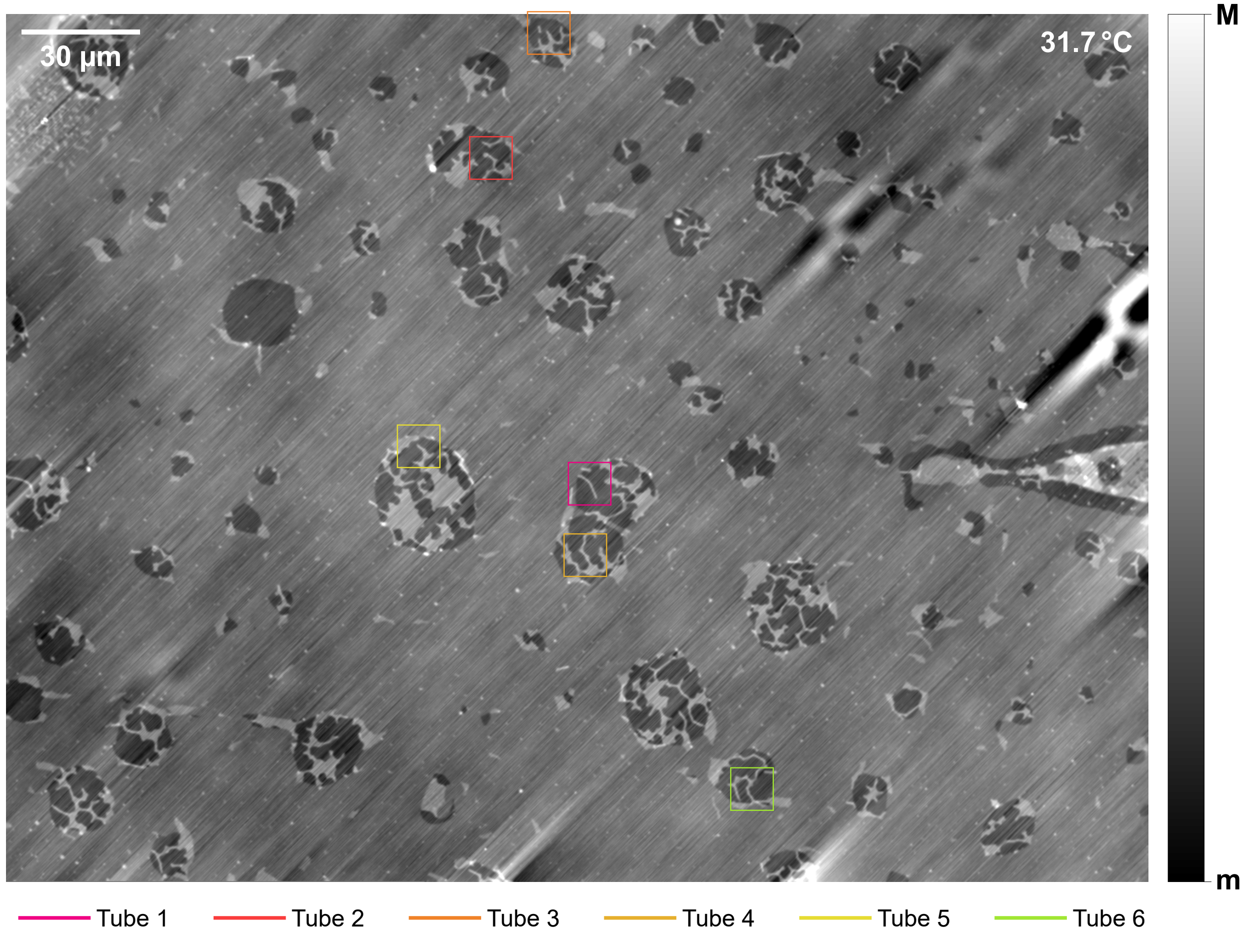

FIG. S14. qDIC phase image at 31.7 °C of the full field of view from which tubes 1 - 6 (measured in Fig. S12) are taken, shown on a scale from $m = -14$ mrad to $M = 14$ mrad.

which S_o - L_d phase coexistence is visible (28.7 °C), and the next temperature point in which the sampled has returned to a homogeneous fluorescence (28.4 °C) is used as the end of the phase transition.

The larger fields of view in which these measurements are taken are shown in Fig. S14 (tubes 1-6) and Fig. S15 (tubes 7-12). Two prominent phase artefacts visible on the upper right hand side of both images are attributed to defocused unwanted features in the beam path, and measurement regions were chosen to avoid proximity to these artefacts. The majority of the sample is unilamellar, with tubes formed from bilamellar patches as described in the main text. Note that the two fields of view shown have similar arrangements; as discussed, this consistency minimises local tension variations that would otherwise obscure the global tension effects resulting from the phase transition. The overall appearance of the

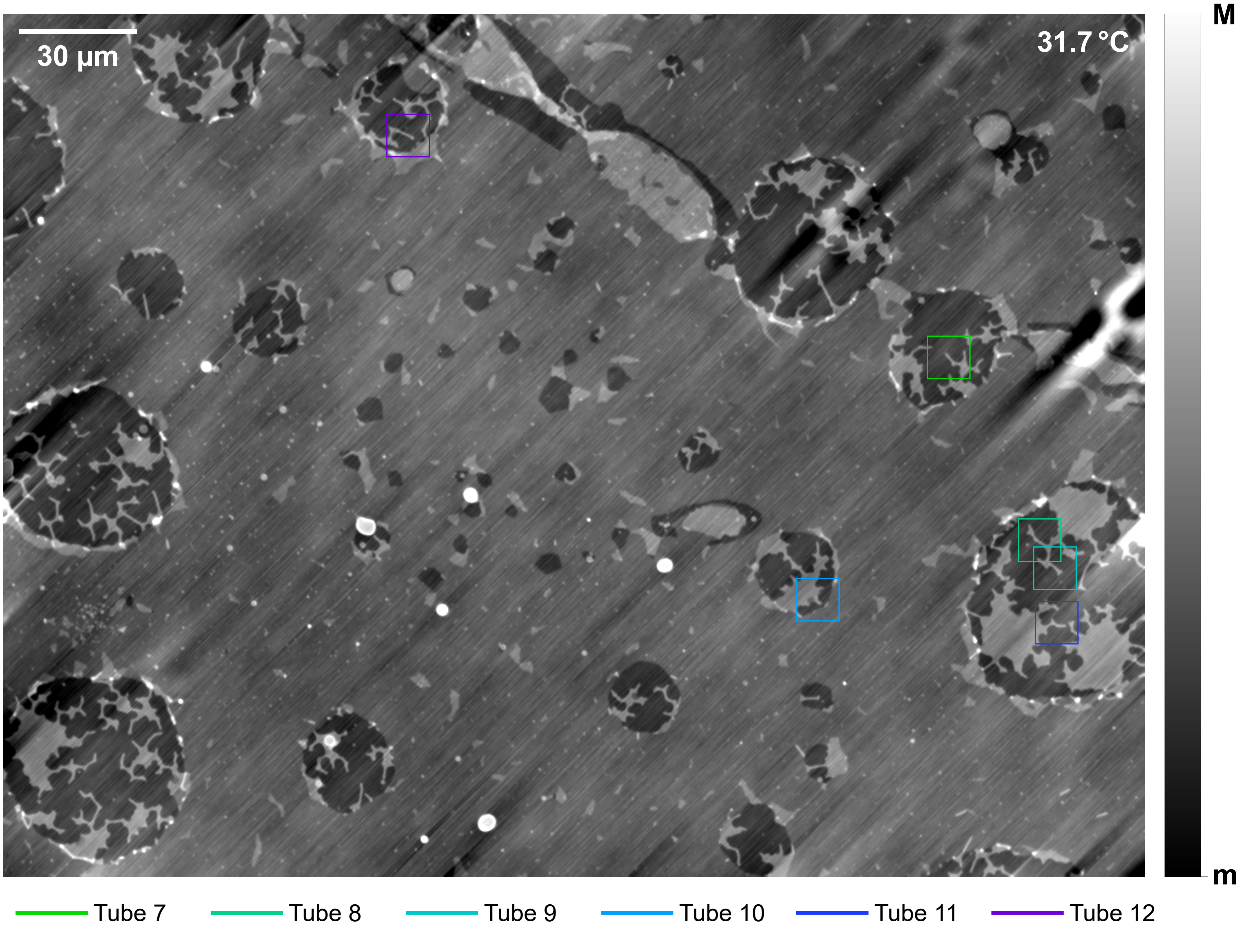

FIG. S15. qDIC phase image at 31.7 °C of the full field of view from which tubes 7 - 12 (measured in Fig. S12) are taken, shown on a scale from $m = -14$ mrad to $M = 14$ mrad.

surface with scattered holes in the unilamellar film decorated by and containing bilamellar structures suggests that they were formed by a rupture and detachment of the surface adhered unilamellar film under tension during the first cooling from the L_d to the S_o phase after hydration. This led to a folding over of the bilayer and resealing into bilamellar regions or edge tubes (see Fig. 4) to avoid unilamellar edges which have a large line energy and thus line tension. The investigated tubes are bilamellar structures in these holes.

The evolution of these tubes during cooling is shown in Fig. S16. The characteristic behaviour of a radius decrease followed by a radius increase can be best observed in the regions of tube 4, 5, 6, and 10, where some of the tubular regions are getting very thin around 30 °C and recover afterwards. As mentioned in the main text, several tubes become disconnected from the larger supported bilayer stack during the cooling; tube 1 for example

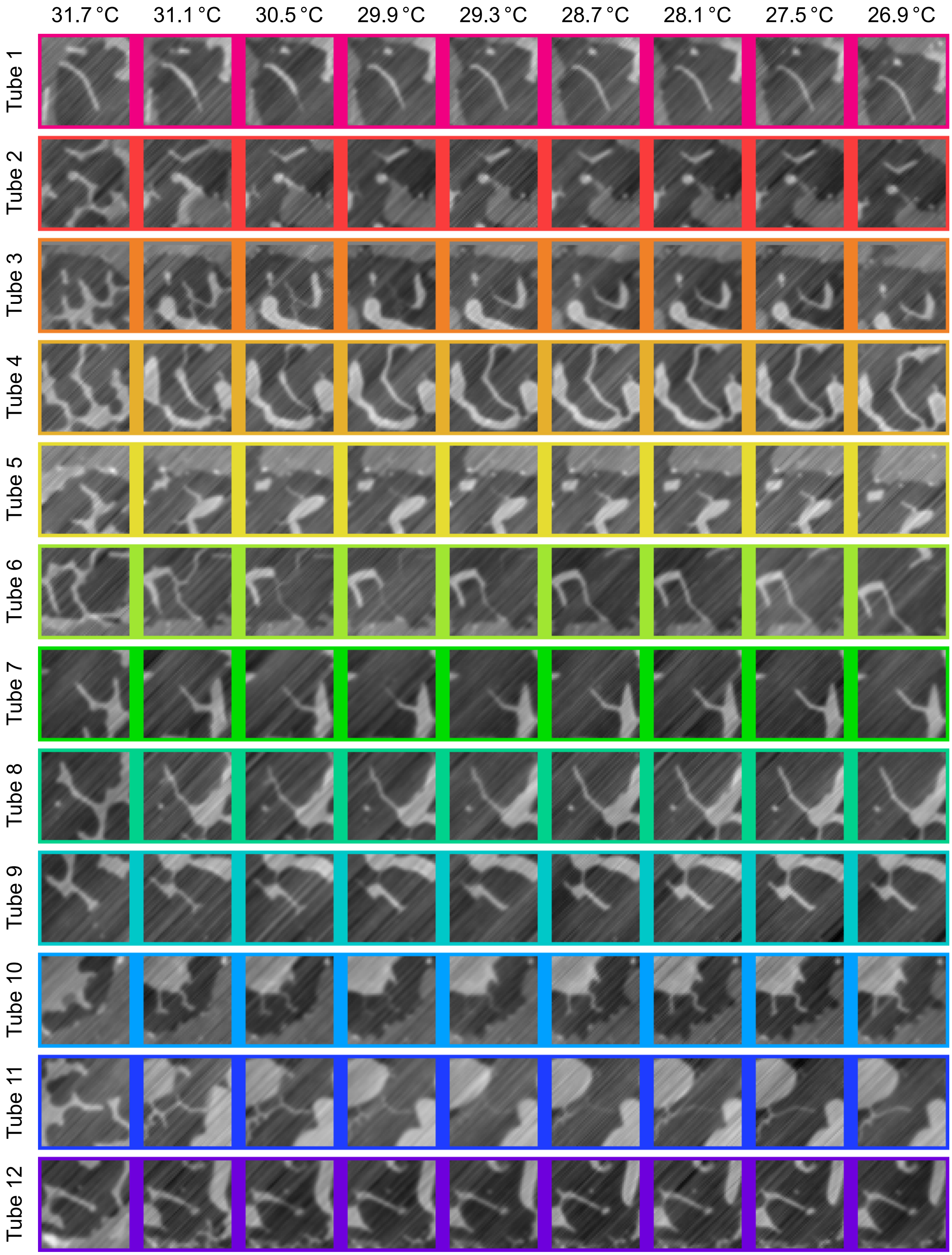

FIG. S16. qDIC phase images of the regions containing each of the twelve tubes analysed in Fig. S12, on a grey scale from −14 mrad to +14 mrad; image size is $10.8 \times 10.8\,\mu m^2$.

becomes visibly disconnected around 30.5 °C. Note that the evolution of the structure of this tube already deviates from that of the general pattern before this point, indicating different tension effects which may contribute to the disconnection. It shows down to 29.9 °C regions of different optical thickness, likely corresponding to coexisting S_o and L_d phase regions along the tube. Similar behaviour can be seen for the V-shaped tube at the top of the image of Tube 2. Tube 2 has a distinct behaviour, exhibiting a structural rearrangement from a tube to a rounded bilamellar patch attached to the edge of a unilamellar region, which has expanded towards what had previously been the end of the tube. This shows the motion of a triple junction between unilamellar and bilamellar regions. Such junctions were observed to be surprisingly stable in position in Fig. 5.

In the main text, we also briefly discuss experiments looking at the cooling of tubes formed on top of planar bilayers. The changes in the integrated phase of these tubes are less consistent than seen in the tubular networks formed on glass, which we attribute to greater variations in their tension. Six representative examples of different behaviour are shown in Fig. S17a, with the evolution of the tube shape during the cooling process shown in Fig. S17b. The cooling rate in this experiments was somewhat faster than that discussed in the main text (approximately -1.2 °C/hour compared to -0.75 °C/hour for the data shown in Fig. 7). The full field of view from which the tubes were taken is shown in Fig. S18. It is a mostly bilamellar region consisting of multiple different patches (the boundaries between bilamellar patches are visible as lines of higher intensity). Tube 1 shows at temperatures of 31.1 °C and below regions of different optical thickness along the tube are seen, likely corresponding to coexisting S_o and L_d phase regions along the tube, of different radii. Tube 3 also shows an interesting behaviour - it is connected to the left bilayer patch and shifts its start position along that edges and rips a gap into the bilayer as the patch is shrinking during cooling. Tube 5 seems to contain internal vesicles, establishing is bilamellar regions, seen as optically thicker regions.

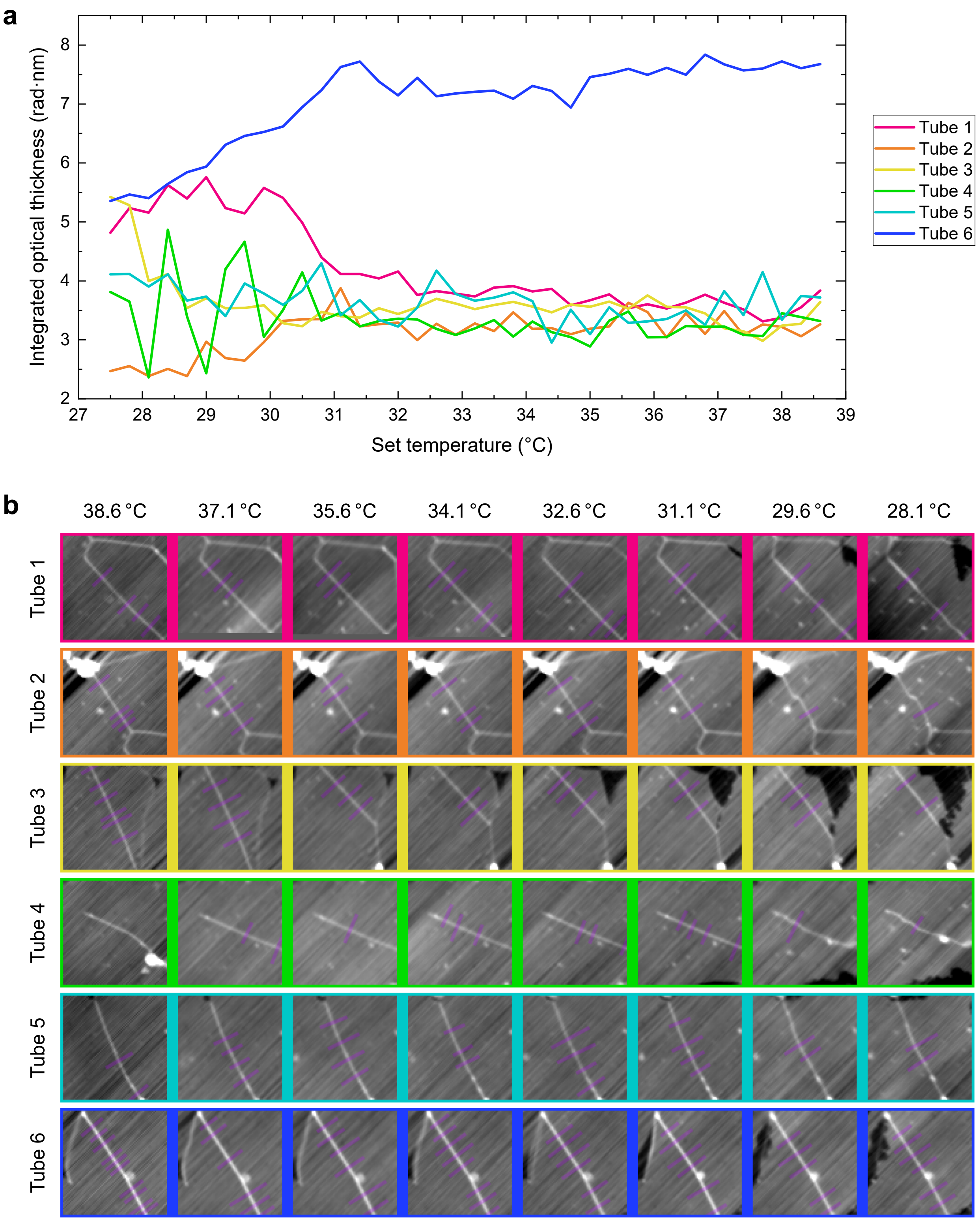

FIG. S17. a) Integrated phase versus temperature for six tubes formed on top of other bilayers; tube 6 appears to be two tubes close together. b) qDIC phase images of each tube in (a), for decreasing temperatures during cooling as indicated (scale from −20 mrad to +10 mrad, image size $16.4 \times 16.4\,\mu m^2$). Positions at which measurements were taken are indicated by purple lines.

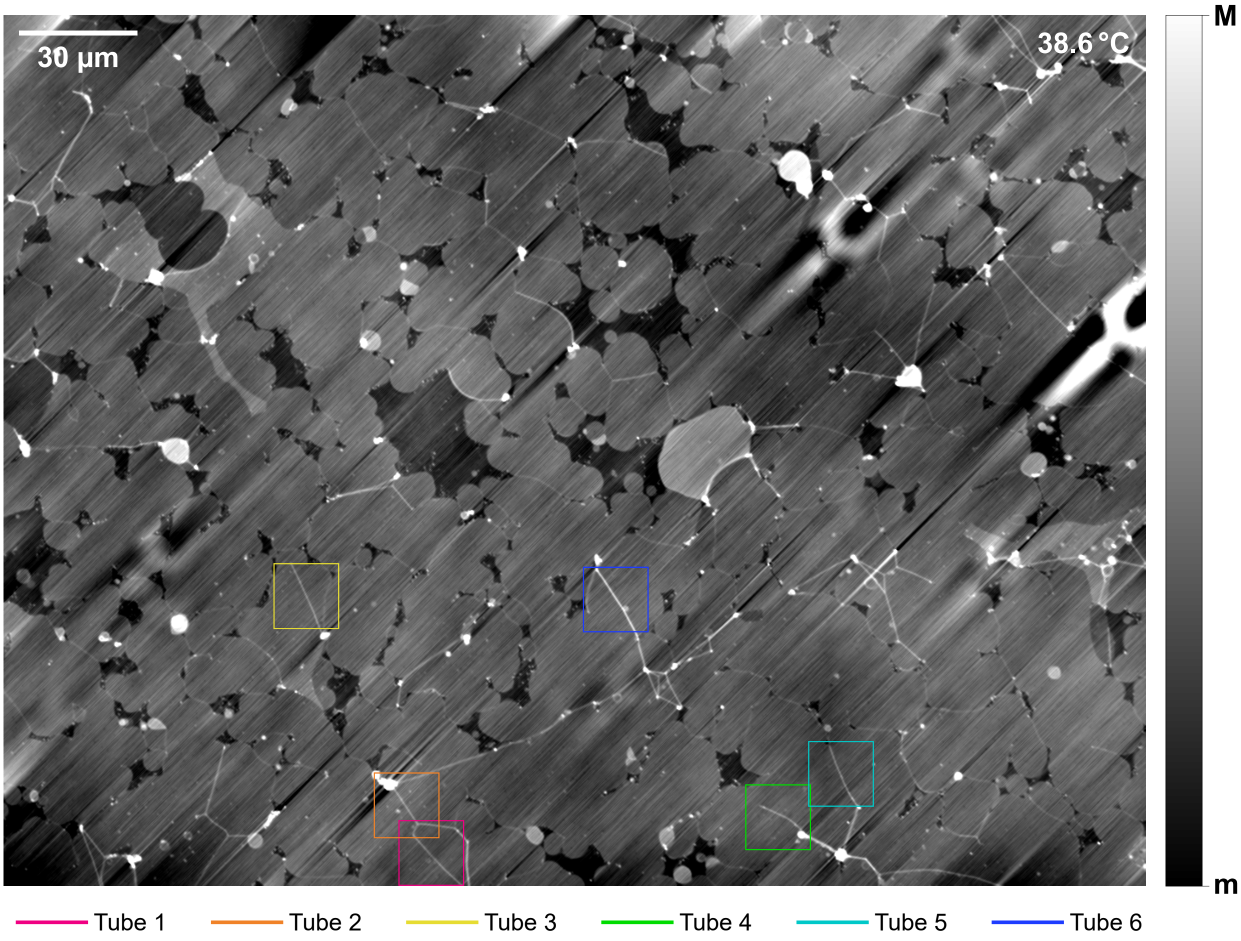

FIG. S18. qDIC phase image of $DC_{15}PC$ lipid bilayers at 38.6 °C of the full field of view from which the tubes measured in Fig. S17 are taken, shown on a scale from $m = -20$ mrad to $M = +10$ mrad. Data for all temperatures are available in the open data for this work.

S8. UNPROCESSED VERSIONS OF QDIC PHASE IMAGES

In Fig. 5 and Fig. S9 we show images of a multilamellar region of a $DC_{15}PC$ bilayer stack in which the different lamellarities are indicated by a colour overlay. In Fig. S19 and Fig. S20 we show the same images without the colour overlay.

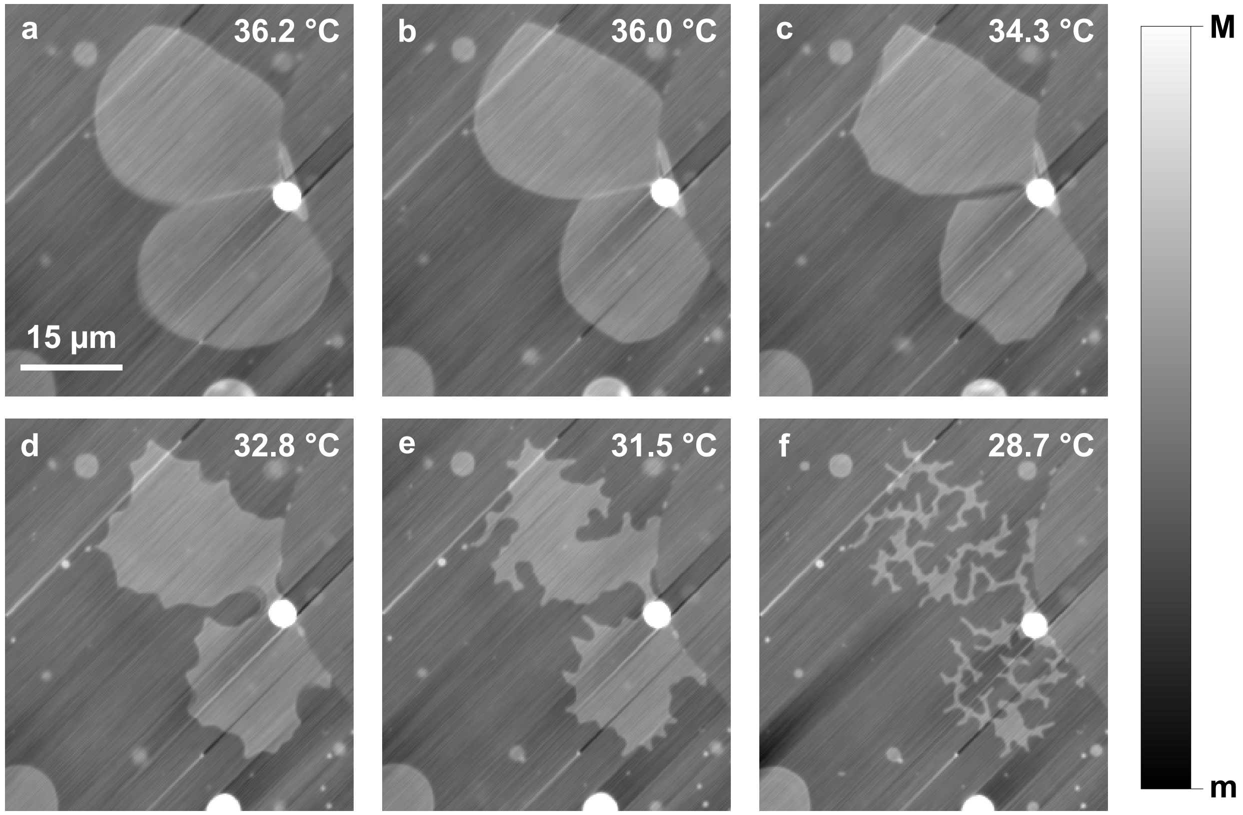

FIG. S19. qDIC phase images of $DC_{15}PC$ lipid bilayers used in Fig. 5 in the main text. Scaled from $m = -3\,\mathrm{mrad}$ to $M = +20\,\mathrm{mrad}$.

The full field of view from which the smaller regions in Fig. 5a-f, Fig. 5i, and Fig. S9 (and correspondingly Fig. S19 and Fig. S20) are taken is shown in Fig. S21.

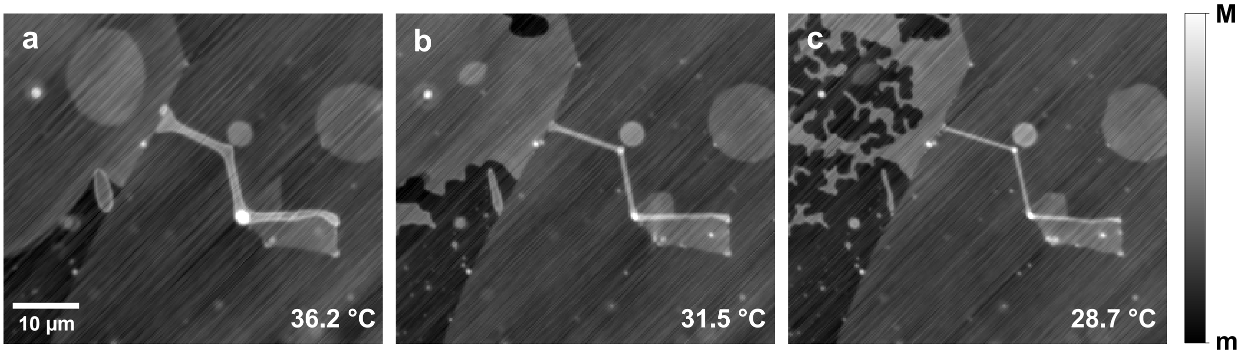

FIG. S20. qDIC phase images of $DC_{15}PC$ lipid bilayers used in Fig. S9 without colour overlay or gradient minimisation. Scaled from $m = -8$ mrad to $M = +24$ mrad.

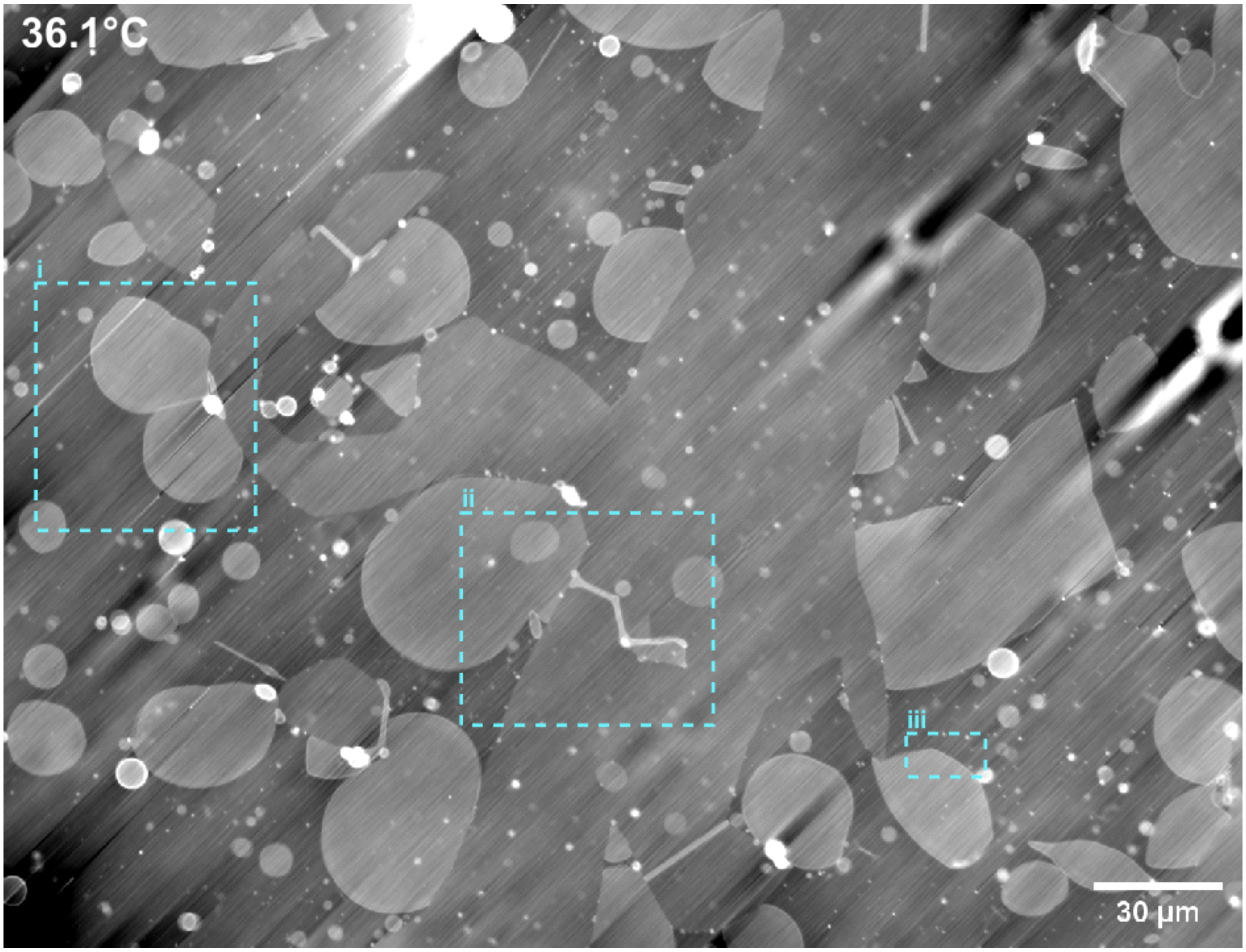

FIG. S21. qDIC phase image of $DC_{15}PC$ lipid bilayers showing the full field of view containing the regions shown in i) Fig. 5a-f and Fig. S19a-f, ii) Fig. S9 and Fig. S20, iii) Fig. 5i, on a scale from -30 mrad to 10 mrad.

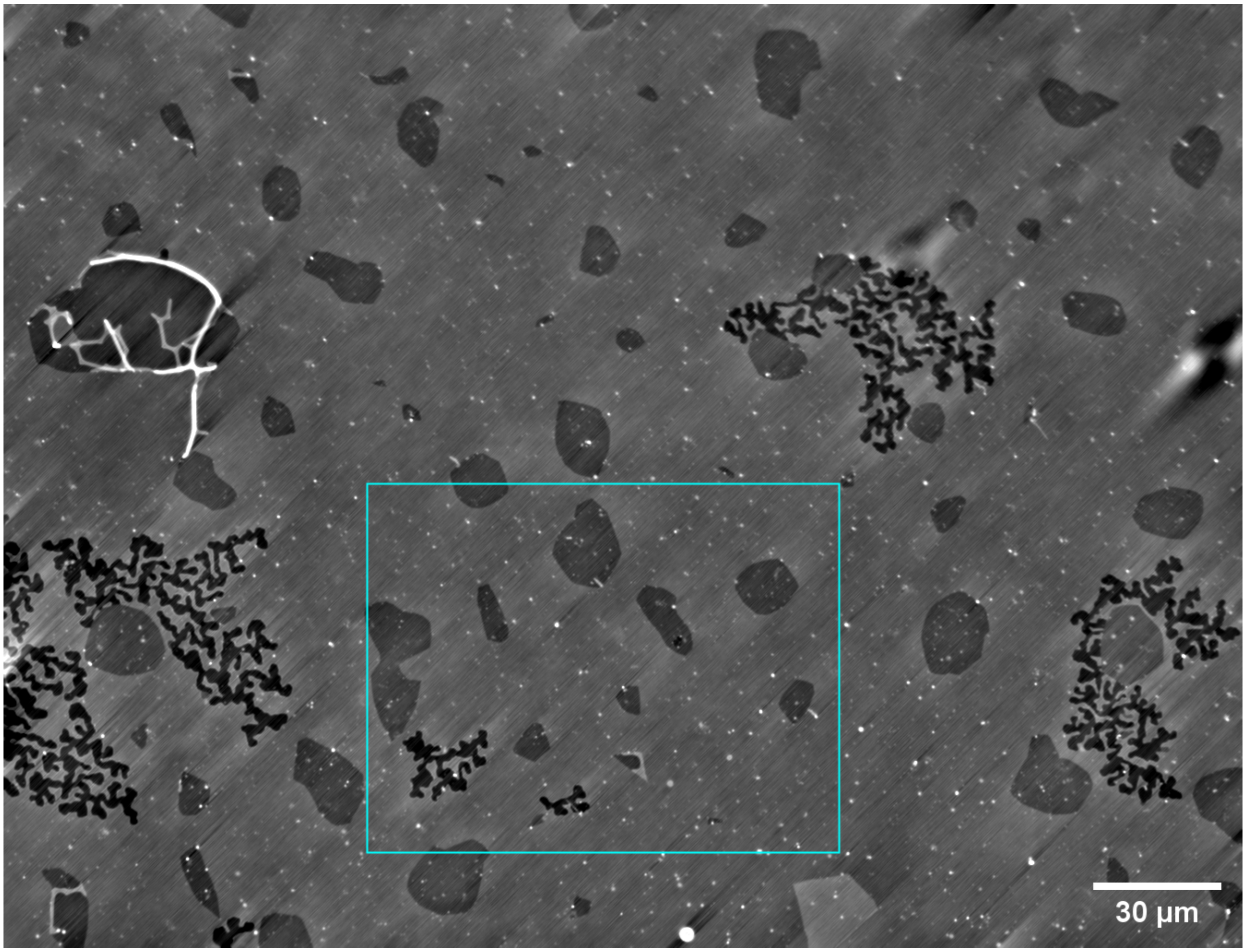

FIG. S22. qDIC phase image of a DOPC bilayer stack in 2 mM Tris, showing the full field of view containing the regions shown in Fig. S4 (indicated by the blue box), on a scale from -10 mrad to 20 mrad.

S9. TUBE MEASUREMENT POSITIONS

Positions of tube profile measurements corresponding to the data shown in Fig. 3 are shown in Fig. S23, Fig. S24, Fig. S25, and Fig. S27 as yellow lines.

A zoomed in section of a double tube structure is shown in Fig. S26. It can be seen that where the two tubes are stacked, the intensity is high and the apparent width is low, while where the two tubes are side-by-side, the intensity is low, but the width is much larger.

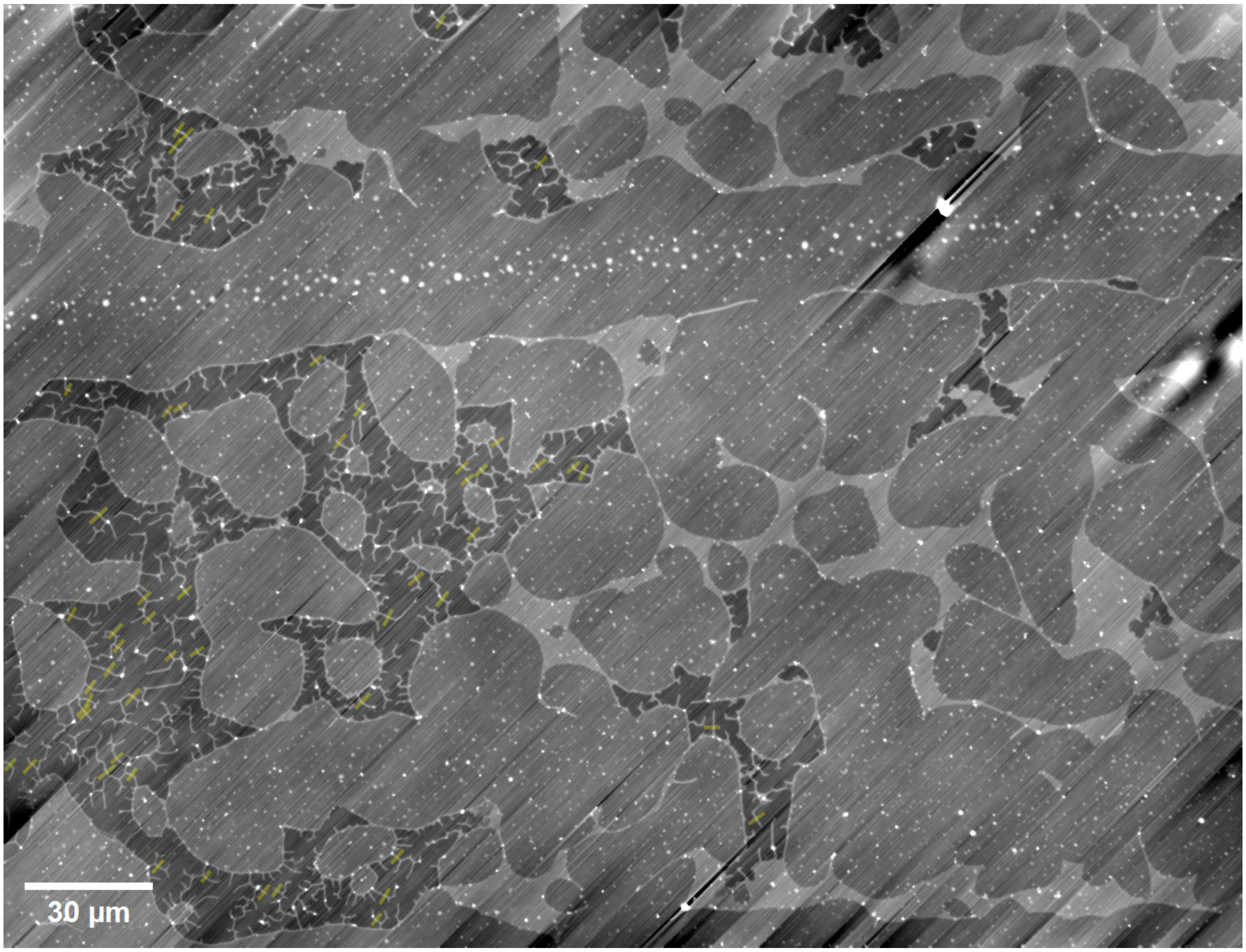

FIG. S23. qDIC phase image of DOPC lipid bilayers with the corresponding tube measurement positions shown in Fig. 3 indicated. Scaled from $m = -15$ mrad to $M = 20$ mrad.

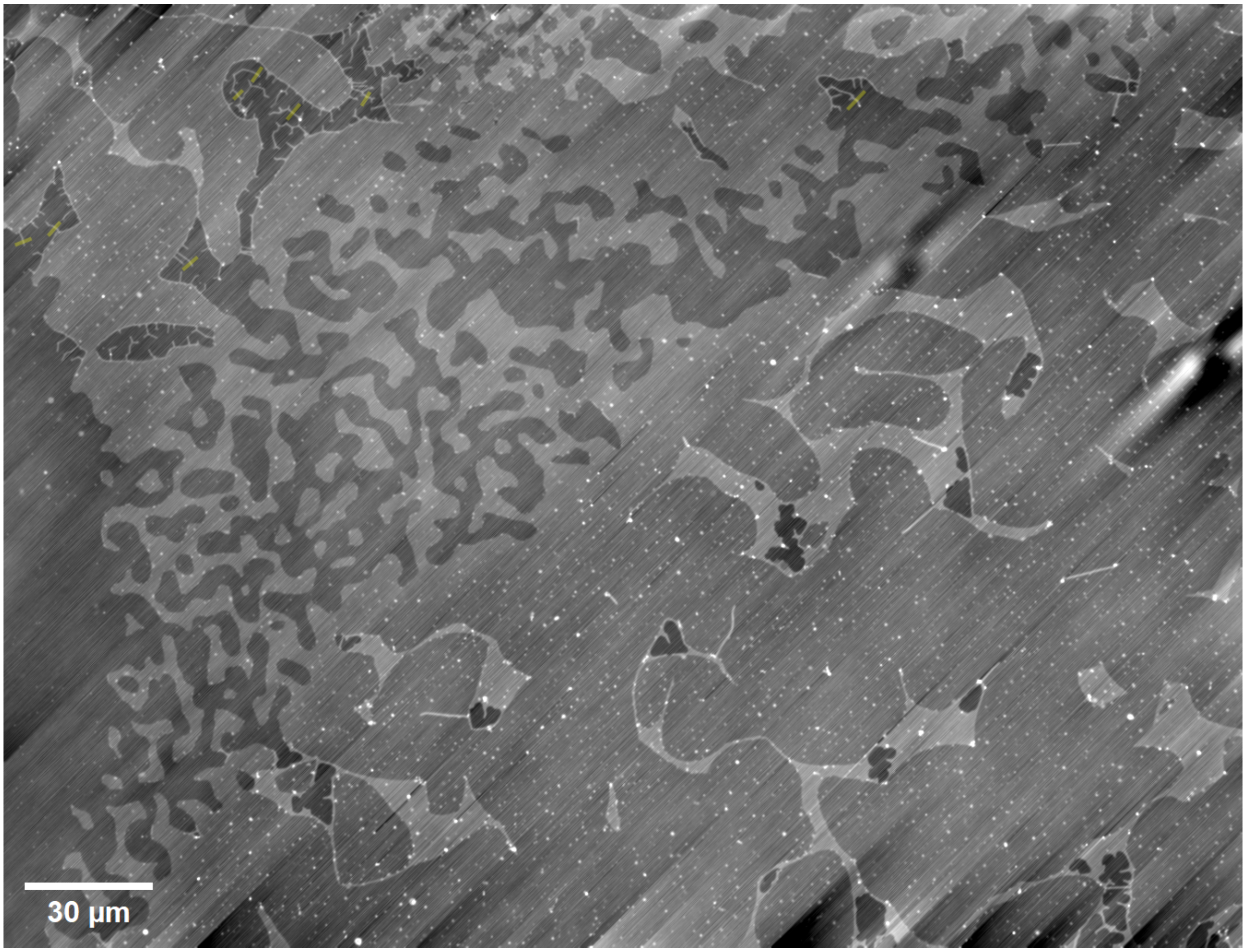

FIG. S24. qDIC phase image of DOPC lipid bilayers with the corresponding tube measurement positions shown in Fig. 3 indicated. Scaled from $m = -15$ mrad to $M = 20$ mrad.

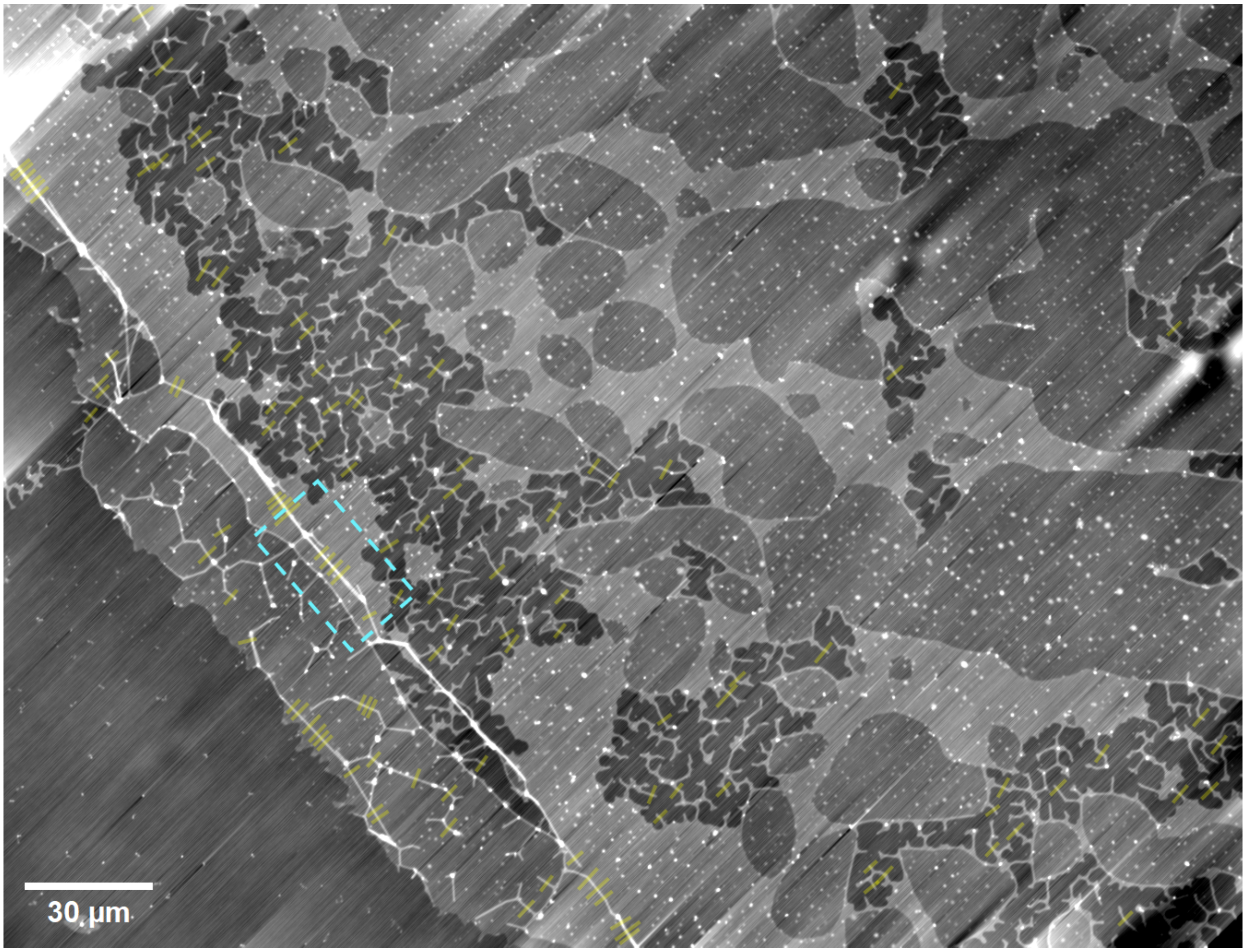

FIG. S25. qDIC phase image of DOPC lipid bilayers with the corresponding tube measurement positions shown in Fig. 3 indicated. The dashed rectangle indicates the region shown in Fig. S26. Scaled from $m = -15\,\mathrm{mrad}$ to $M = 20\,\mathrm{mrad}$.

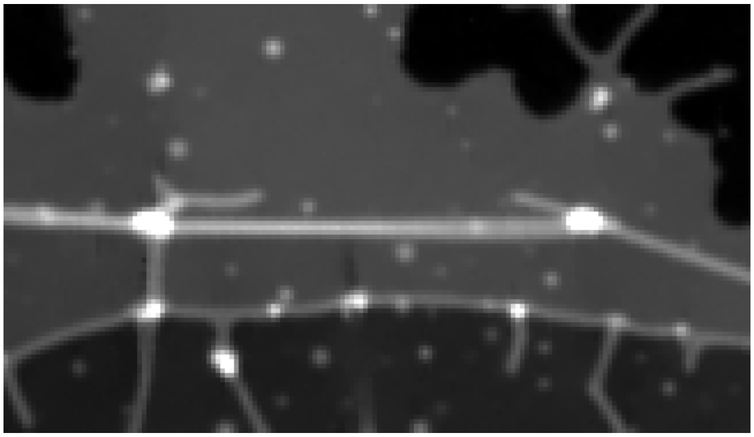

FIG. S26. Minimised qDIC phase image of a double tube structure of DOPC lipid bilayers, of the region of 35 µm width indicated in Fig. S25 as dashed rectangle. Scaled from $m = 0$ mrad to $M = 40$ mrad. The decrease of the width and concomitant increase of the amplitude of the horizontal line connecting the two point-like structures can be interpreted as two tubes starting side by side at the right vesicular structure and ending up stacked at the left vesicular structure.

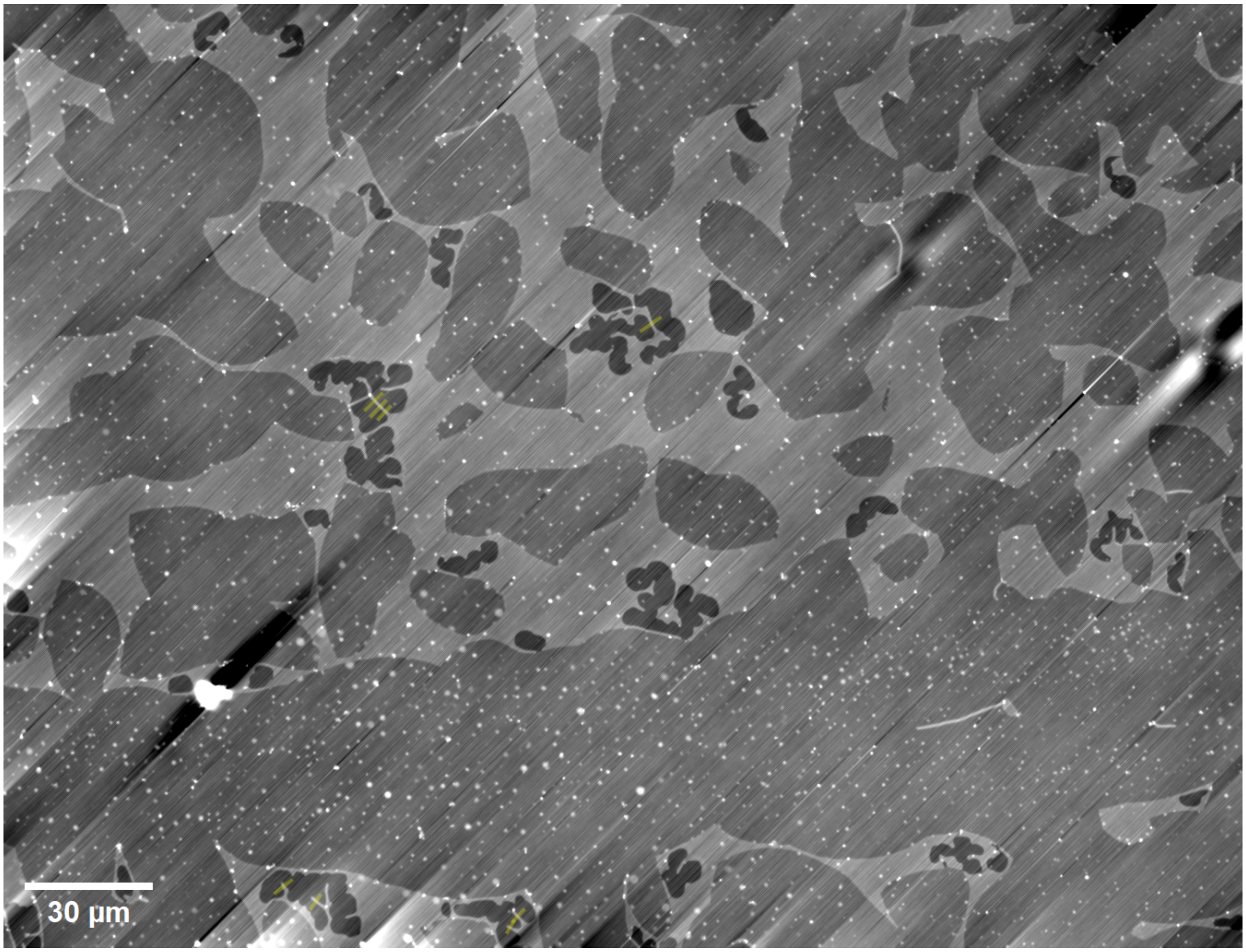

FIG. S27. qDIC phase image of DOPC lipid bilayers with the tube measurement positions indicated. Scaled from $m = -15\,\text{mrad}$ to $M = 20\,\text{mrad}$.

[1] David Regan, Joseph Williams, Paola Borri, and Wolfgang Langbein. Lipid bilayer thickness measured by quantitative dic reveals phase transitions and effects of substrate hydrophilicity. *Langmuir*, 35(43):13805–13814, sep 2019. doi:10.1021/acs.langmuir.9b02538.

[2] Savitha Devanathan, Zdzislaw Salamon, Göran Lindblom, Gerhard Gröbner, and Gordon Tollin. Effects of sphingomyelin, cholesterol and zinc ions on the binding, insertion and aggregation of the amyloid Aβ1-40 peptide in solid-supported lipid bilayers. *FEBS J.*, 273(7): 1389–1402, 2006. ISSN 1742-4658. doi:10.1111/j.1742-4658.2006.05162.x.

[3] Samuel Hamilton, David Regan, Lukas Payne, Wolfgang Langbein, and Paola Borri. Sizing individual dielectric nanoparticles with quantitative differential interference contrast microscopy. *Analyst*, 147:1567–1580, 2022. doi:10.1039/D1AN02009A. URL `http://dx.doi.org/10.1039/D1AN02009A`.

[4] Dominik Drabik, Grzegorz Chodaczek, Sebastian Kraszewski, and Marek Langner. Mechanical properties determination of dmpc, dppc, dspc, and hspc solid-ordered bilayers. *Langmuir*, 36 (14):3826–3835, Apr 2020. ISSN 0743-7463. doi:10.1021/acs.langmuir.0c00475.
